# Relative efficacy of lasmiditan versus rimegepant and ubrogepant as acute treatments for migraine: network meta-analysis findings

**DOI:** 10.1186/s10194-022-01440-w

**Published:** 2022-07-06

**Authors:** Pepa Polavieja, Mark Belger, Shiva Kumar Venkata, Stefan Wilhelm, Erin Johansson

**Affiliations:** 1 Eli Lilly and Company, Indianapolis, Indiana USA; 2 Avenida de la Industria 30, 28108 Alcobendas, Madrid Spain

**Keywords:** Acute treatments, Efficacy, Gepants, Lasmiditan, Rimegepant, Ubrogepant, Migraine, Network meta-analysis

## Abstract

**Background:**

In the absence of head-to-head trials, comprehensive evidence comparing onset of efficacy of novel agents for acute treatment of migraine is lacking. This study aimed to explore the relative efficacy of lasmiditan (serotonin [5-hydroxytryptamine] 1F receptor agonist) versus rimegepant and ubrogepant (calcitonin gene-related peptide antagonists) for the acute oral treatment of migraine through network meta-analysis (NMA).

**Methods:**

Data included in the NMA were identified through a systematic literature search (conducted April 2018, updated May/December 2020) of phase II–IV, randomised controlled trials (RCTs) in adults with chronic/episodic migraine with/without aura. Treatments included: lasmiditan 50, 100, 200 mg; rimegepant 75 mg; ubrogepant 25, 50, 100 mg. Pairwise treatment comparisons from Bayesian fixed-effect/random-effects NMA, adjusted by baseline risk where appropriate, were conducted. Comparisons were reported as odds ratios with 95% credible intervals. Early-onset efficacy endpoints included: pain freedom at 2 hours and pain relief at 1 and 2 hours. Adverse drug reaction (ADR) profiles were summarised. Heterogeneity and inconsistency in the network were explored; sensitivity analyses investigated robustness of findings.

**Results:**

Across 12 RCTs included in the base case, females represented >80% of included patients (mean age 37.9–45.7 years). Odds of achieving both pain freedom and pain relief at 2 hours were higher with lasmiditan 100 and 200 mg versus rimegepant 75 mg and ubrogepant 25 and 50 mg. Results for pain relief at 1 hour were consistent with those at 2 hours, but fewer comparisons were available. There were no statistically significant differences between lasmiditan 50 mg and ubrogepant or rimegepant for any outcome. Sensitivity analyses were in the same direction as base case analyses. Most commonly reported ADRs (incidence ≥2%) were: dizziness, fatigue, paraesthesia, sedation, nausea/vomiting and muscle weakness with lasmiditan; nausea with rimegepant; and nausea, somnolence and dry mouth with ubrogepant.

**Conclusions:**

The efficacy findings of this indirect comparison indicate that lasmiditan 100 mg or 200 mg might be an appropriate acute treatment option for patients with migraine seeking a fast onset of action. Differently from rimegepant and ubrogepant, lasmiditan use is associated with mainly neurological events, which are mostly mild or moderate in severity and self-limiting.

350/350 words

**Supplementary Information:**

The online version contains supplementary material available at 10.1186/s10194-022-01440-w.

## Background

Migraine is a highly prevalent common primary headache disorder with a high associated socioeconomic and patient-level burden [1, 2]. In 2016, migraine was the second leading cause of years lived with disability worldwide, after low back pain [3].

Pharmacological management options for migraine include acute treatment, emergency treatment and preventive treatment [4]. Acute treatments for migraine aim to achieve rapid and sustained freedom from pain and other migraine-associated symptoms, restore functional ability and minimise the use of rescue medication, repeat doses and healthcare resources, and the occurrence of adverse events (AEs) [5]. Triptans are considered the current standard of care for the acute treatment of migraine attacks of moderate to severe severity [6]; however, the vasoconstrictive properties of triptans preclude their use in patients with underlying cardiovascular diseases or those at risk of certain adverse cardiovascular events [7, 8]. Additionally, although beneficial in some people, many patients exhibit insufficient efficacy and/or tolerability to triptan therapy, and hence have a high unmet need for an effective acute treatment for migraine [9]. A US longitudinal population-based study (American Migraine Prevalence and Prevention) found that, of more than 5500 people with episodic migraine, 41% reported having at least one unmet treatment need with their current acute treatment, which included dissatisfaction with treatment efficacy and safety [10].

The fact that triptans are contraindicated in some patients with cardiovascular disease [11] led to the development of the first-in-class ditan, lasmiditan. Lasmiditan is a centrally penetrant, high-affinity, highly selective serotonin (5-hydroxytryptamine) 1F receptor (5-HT_1F_) agonist that exerts its therapeutic effects by blocking activation of the trigeminal neurones, thus inhibiting migraine attack pain pathways, without causing vasoconstriction in human coronary arteries [12].

Recently, other acute treatments for migraine have also become available. The gepants rimegepant and ubrogepant are orally administered antagonists of the calcitonin gene-related peptide (CGRP) receptor that competitively block the effects of CGRP [13, 14].

Although lasmiditan [15–19], rimegepant [20–23] and ubrogepant [24–27] have all shown efficacy as acute treatments for migraine in a range of placebo-controlled randomised controlled trials (RCTs), direct comparisons in the form of head-to-head RCTs are lacking. In the absence of such data, network meta-analysis (NMA) offers a way of comparing interventions simultaneously in a single analysis. To date, three NMAs have been published, comparing the efficacy of lasmiditan, rimegepant and ubrogepant – those of Johnston et al. 2022 [28], Agboola et al. 2020 [29] and Yang et al. 2021 [30]. Since publication of these NMAs, new key data/evidence for lasmiditan have become available from the registration studies CENTURION [17] and MONONOFU [18]. The aim of this study was, therefore, to explore the relative efficacy of lasmiditan compared to both rimegepant and ubrogepant for the acute treatment of migraine through an NMA including the most up-to-date evidence available and to explore early onset endpoints.

## Methods

### Systematic literature review

A general systematic literature review (SLR) was carried out to identify phase II–IV RCTs of any acute medication used for the treatment of patients with chronic or episodic migraine with or without aura. Conduct of the SLR was compliant with guidelines provided by the Cochrane Collaboration, Preferred Reporting Items for Systematic Reviews and Meta-Analyses (PRISMA) [31] and the Centre for Reviews and Dissemination [32].

The original literature search was conducted on 4 April 2018 and updated using the same methodology on 26 May and 15 December 2020. A more recent search of the literature, conducted on 31 August 2021, identified no additional studies. Searches in MEDLINE®, MEDLINE® In-process, Epubs ahead of print, Embase and the Cochrane Central Register of Controlled Trials via the OVID SP® search engine were conducted using search strategies specific to each database (see Supplementary Table 1). Additional searches were conducted of conference abstracts presented at the American Headache Society, International Headache Society, American Academy of Neurology and the European Headache Federation (2019–2020), and of Clinicaltrials.gov and the World Health Organization International Clinical Trials Registry Platform Search Portal to identify ongoing trials (December 2020).

Eligibility criteria for inclusion in the SLR are summarised in Supplementary Table 2. Non-RCTs and publications in any language other than English were excluded. Study abstracts and full-text articles were reviewed according to the eligibility criteria by two independent systematic reviewers, with any queries being referred to a third reviewer. Data (including study characteristics, patient characteristics, efficacy outcomes data and safety outcomes) were extracted and independently checked. In an effort to reduce publication bias, data reported only in figures in included articles were digitally extracted using WebPlotDigitizer [33]. Risk of bias assessments (including randomisation and concealment allocation methods, description and method of blinding [participants, care providers and outcome assessors], incomplete outcome data and selective reporting [not possible for conference abstracts due to text limitations]) were conducted and reported for the studies included in the NMA.

### NMA

All analyses were conducted using R (version 3.2.2) and JAGS (version 3.4) to perform the Markov Chain Monte Carlo sampling to fit Bayesian NMA.

For the purpose of conducting the current NMA, which aimed to explore specifically the relative efficacy of lasmiditan, rimegepant and ubrogepant for the acute treatment of migraine, RCTs identified in the SLR were further selected if they satisfied the NMA-specific population, intervention, comparator and outcome selection (PICOS) statement Table 1.

**Table 1:** Population, intervention, comparator and outcome selection (PICOS) criteria

Population	Adults (aged (≥18 years) with episodic or chronic migraine with or without aura (where specified, International Headache Society diagnostic criteria, Headache Classification Committee of the International Headache Society, 2013 [34])
Intervention/comparators	Lasmiditan (50 mg, 100 mg, 200 mg) Rimegepant (75 mg) Ubrogepant (25 mg, 50 mg, 100 mg)
Outcomes	• Pain freedom at 2 hours (pain reduced from moderate or severe to none without use of rescue medication within 2 hours) • Pain relief at 2 hours (pain reduced from moderate or severe to none or mild without use of rescue medication within 2 hours) • Pain relief at 1 hour (pain reduced from moderate or severe to none or mild without use of rescue medication within 1 hour) • MBS freedom at 2 hours (freedom from the MBS, as identified by the patient, from the associated symptoms of nausea, phonophobia and photophobia) • Sustained pain freedom over 24 hours (pain freedom at 2 hours, sustained for 24 hours, without the use of rescue medication or a second dose of study medication) • Discontinuation due to AEs
Study design	Randomised controlled trials

*AE* adverse event, *MBS* most bothersome symptom

Bayesian hierarchical NMA was used to estimate differences in efficacy between lasmiditan and each of the gepants, rimegepant and ubrogepant, and was conducted in accordance with guidelines set by the National Institute for Health and Clinical Excellence Decision Support Unit [35]. Discontinuation due to AEs was chosen for estimating differences in safety; however, due to the finding that all studies either reported no information or zero events in all treatment arms (with the exception of one event for lasmiditan 100 mg in the SPARTAN study [2]), models for discontinuation due to AEs had poor fit and no quantitative comparative assessment could be performed. The adverse drug reaction (ADR) profiles of the three agents according to the US prescribing information were therefore summarised.

The base case model of the NMA consisted of all relevant RCTs identified following application of the PICOS statement Table 1 to studies identified in the SLR. Fixed-effect and random-effects models were performed for each endpoint. Choice of model (fixed effect or random effect; Supplementary Table 3) for each analysis was evaluated on the model fit as measured by: deviance information criteria; assessment of residual deviance; convergence of the models; whether there were sufficient data to inform the random effects between-study variance; and whether there was evidence that the random effects prior dominated the posterior simulations indicating that there was insufficient heterogeneity in the data to inform this additional parameter. Convergence for all models was assessed using trace plots as modified by Brooks et al. [36].

Heterogeneity was explored visually by inspecting the magnitude and variability of the study results within each forest plot and by evaluating the inconsistency parameter (*I*^2^), the between studies variance, and the heterogeneity statistic Q. Due to differences in the placebo response across trials and its potential impact on treatment effect [37], models (e.g., those for the pain freedom outcomes) were adjusted for baseline risk (i.e., placebo response) when appropriate.

Pairwise treatment comparisons were conducted in accordance with published guidelines [35]. Comparisons were reported as odds ratios (ORs) with 95% credible interval (Crl) (OR >1 indicated greater odds that findings favoured the lasmiditan arm over the comparator arm for each endpoint). 95% CrI that did not include 1.00 were considered to show a statistical difference. As all the outcomes assessed were binary, a binomial distribution was assumed.

Surface under the cumulative ranking curve (SUCRA) values [38] were used to capture any uncertainty in the estimates by taking into account the full area under the ranking curves. SUCRA values range from 0% to 100%, where 100% represents the certainty that a treatment is the best of those analysed and 0% represents the certainty that a treatment is the worst.

For each Bayesian Markov Chain Monte Carlo three chains were used, each comprising 480,000 samples after discarding 80,000 samples as burn-in and thinning by a rate of 24. The number of samples was doubled when there was evidence of non-convergence or autocorrelation. The initial values for these parameters and each chain were chosen by selecting random samples from a normal distribution with mean 0 and variance 1.

For the relative treatment effects and study-specific effects, *μ*_i1_ and *δ*_i1k_*I*_{k>1}_, a normal distribution with mean 0 and variance 10.000 was chosen, N(0,100^2^). For the between-study variance, ${\sigma}_{\delta}^2,$ an uninformative uniform of parameters 0 and 2 was placed, U [0,2]. This distribution assumes that any value between 0 and 2 is equally likely to represent the between-study standard deviation in the treatment effects. An informative prior for the between-study variance was tested in case there was indication of non-convergence of the models (${\sigma}_{\delta}^2$ ~ LN(-2.06, 1.51^2^) (where LN is the lognormal distribution) [39, 40], but was not found to improve convergence.

A series of sensitivity analyses were performed depending on the availability of data within the networks and chosen base case analysis:

- Sensitivity analysis 1 – including only phase III trials.
- Sensitivity analysis 2 – analysing rimegepant according to its mode of administration (tablet or oral disintegrating tablet [ODT]).
- Sensitivity analysis 3 – exploration of very early-onset pain freedom (at 30 min and 1 hour), pain relief (at 30 min) and most bothersome symptom (MBS) freedom at 1 hour.

## Results

**Fig. 1:**
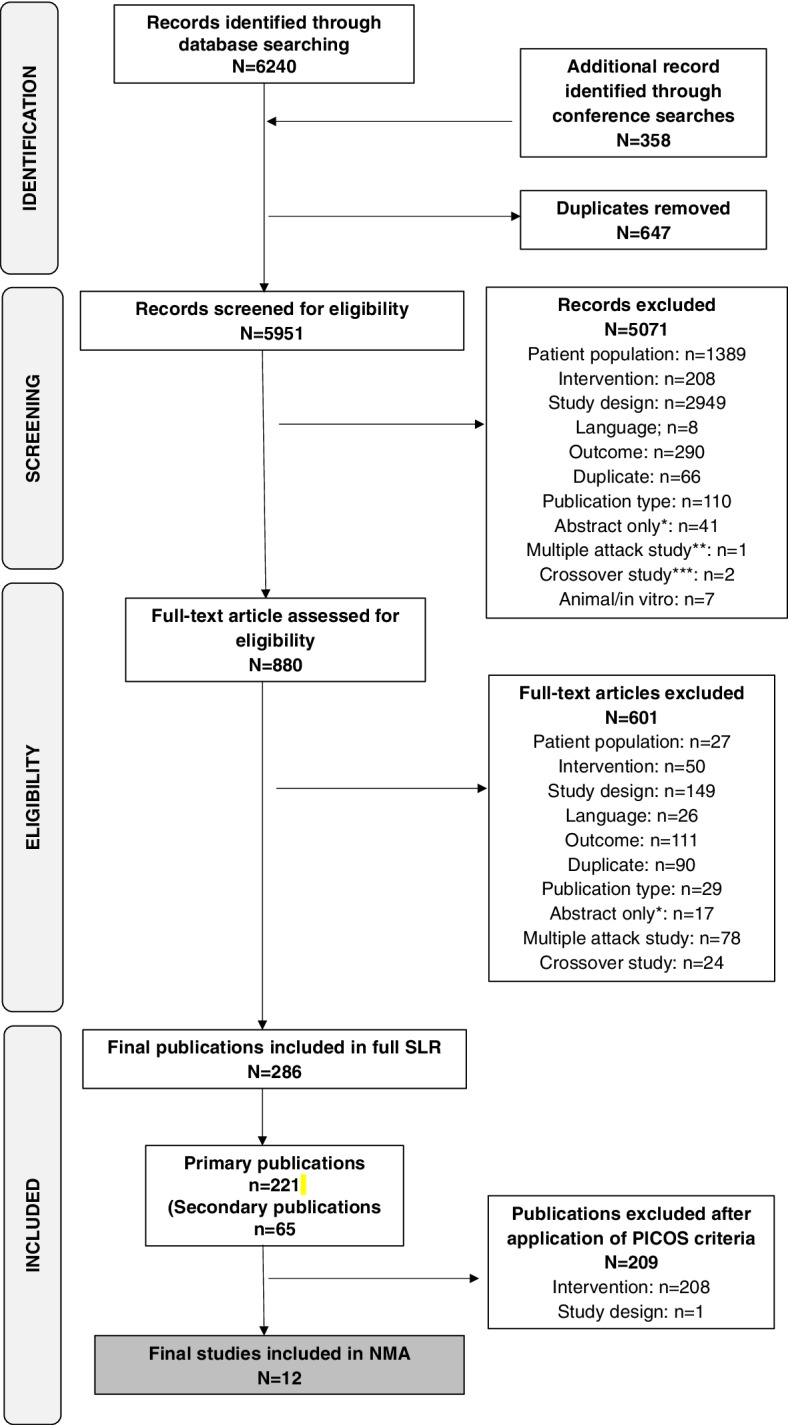
PRISMA diagram representing the studies included in different stages of the SLR and current NMA. The SLR was first run in April 2018, and updated in May 2020 and December 2020. A more recent search of the literature, conducted on 31 August 2021, identified no additional studies. *Applies only to abstracts screened for full-text articles. A few prespecified specific conference abstracts and years were screened separately; therefore, conference abstracts and articles for which only information in abstract form were excluded from the main screening. **Abstract was a multiple attack study for which information was available only in aggregated form. ***Crossover study was a conference abstract for which aggregated data only were available. ^†^No data were extracted from secondary publications. NMA, network meta-analysis; PICOS, population, intervention, comparator and outcome selection; PRISMA, Preferred Reporting Items for Systematic Reviews and Meta-Analyses; SLR, systematic literature review

Across the SLR (original search, and May and December 2020 updates), a total of 6240 records were identified for review. An additional 358 records were identified through the search of conferences/registries. After 647 duplicates were removed, 5951 records were reviewed for inclusion, from which 5071 were excluded after screening. After full-text review of 880 records, 286 publications detailing 221 primary publications and 65 secondary publications were identified having met the inclusion criteria for the SLR. Application of PICOS criteria for the current NMA Table 1 resulted in exclusion of a further 209 publications. Hence, a total of 12 primary publications (detailing 12 studies) (including 3 from the original SLR, and 7 and 2, respectively, from the May and December 2020 updates) were included in the NMA (Fig. 1). Table 2 provides information on the interventions and outcomes assessed in the NMA, for each of the included studies. All the included studies were published between 2012 and 2020, and study designs were similar and consistent with then current clinical guidelines (see Supplementary Table 4 for more information). Baseline patient characteristics were comparable between the studies Table 3. Across all studies, females represented over 80% of the included patients and mean age ranged from 37.9 to 45.7 years.

**Table 2:** Included studies: interventions and outcomes assessed in the NMA

Studies included	Interventions studied and assessed in the NMA	Pain freedom at 2 hours	Pain relief at 2 hours	Pain relief at 1 hour	MBS freedom at 2 hours	Sustained pain freedom over 24 hours	Pain freedom at 30 min	Pain freedom at 1 hour	MBS freedom at 1 hour	Pain relief at 30 min
Lasmiditan vs placebo										
SAMURAI Kuca et al. 2018 [15]	Lasmiditan 100 mg, 200 mg	✓	✓	✓	✓	✓	✓	✓	✓	✓
SPARTAN Goadsby et al. 2019 [16]	Lasmiditan 50 mg, 100 mg, 200 mg	✓	✓	✓	✓	✓	✓	✓	✓	✓
CENTURION Ashina et al. 2021 [17]	Lasmiditan 100 mg, 200 mg	✓	✓	✓	✓	✓	✓	✓	✓	✓
MONONOFU Sakai et al. 2021 [18]	Lasmiditan 50 mg, 100 mg, 200 mg	✓	✓	✓	✓	✓	✓	✓	✓	✓
Färkkilä et al. 2012 [19]	Lasmiditan 50 mg, 100 mg, 200 mg	✓	✓							
Rimegepant vs placebo										
Study 301 Lipton et al. 2018 [20]	Rimegepant 75 mg tablets	✓	✓		✓	✓				
Study 302 Lipton et al. 2019 [21]	Rimegepant 75 mg tablets	✓	✓		✓	✓				
Study 303 Croop et al. 2019 [22]	Rimegepant 75 mg ODT	✓	✓	✓	✓	✓				✓
Marcus et al. 2014 [23]	Rimegepant 75 mg tablets	✓	✓			✓				
Ubrogepant vs placebo										
ACHIEVE I Dodick et al. 2019 [24]	Ubrogepant 50 mg, 100 mg	✓	✓		✓	✓				
ACHIEVE II Lipton et al. 2019 [25]	Ubrogepant 25 mg, 50 mg	✓	✓		✓	✓				
Voss et al. 2016 [26]	Ubrogepant 25 mg, 50 mg, 100 mg	✓	✓			✓				
ACHIEVE I + ACHIEVE II pooled data Goadsby et al. 2021 [27]	Ubrogepant 50 mg	✓		✓	✓		✓	✓	✓	✓

Bold ticks denote the primary endpoint(s) of the study

*MBS* most bothersome symptom, *NMA* network meta-analysis, *ODT* oral disintegrating tablet

**Table 3:** Included studies: baseline patient characteristics

	Lasmiditan vs placebo					Rimegepant vs placebo				Ubrogepant vs placebo		
Characteristic	SPARTAN Kuca et al. 2018 [15]	SAMURAI Goadsby et al. 2019 [16]	CENTURION Ashina et al. 2021 [17]	MONONOFU Sakai et al. 2021 [18]	Färkkilä et al. 2012 [19]	Study 301 Lipton et al. 2018 [20]	Study 302 Lipton et al. 2019 [21]	Study 303 Croop et al. 2019 [22]	Marcus et al. 2014 [23]	ACHIEVE I Dodick et al. 2019 [24]	ACHIEVE II Lipton et al. 2019 [25]	Voss et al. 2016 [26]
N^a^ (baseline)	1856	2583	1471	691	321	1162^b^	1072	1351	320	1436	1465	425
Female, n (%)	1552 (83.6)	2174 (84.2)	1236 (84)	574 (83.1)	277 (86.3)	NA (85.5)	951 (88.7)	1147 (84.9)	277 (86.6)	1266 (88.2)	1317 (89.9)	372 (87.5)
Mean^c^ age, years (range)^d^	41.4–42.4	41.8–43.4	41.0–42.0	44.7–45.7	39.5–42.0	41.6^d^	40.2–40.9	40.0–40.3	37.9–38.5	40.1–40.9	41.2–41.7	40.5–41.9
Mean^c^ duration of disease, years (range)^d^	18.9–19.7	17.6–19.2	NA	23.7–24.7	NA	NA	NA	NA	NA	17.9–19.1	18.1–19.2	NA

^a^Data are from patients receiving placebo or the active treatments included in the NMA; ^b^Number randomised; ^c^Mean for total study population; ^d^Range is the range of mean values across each treatment group of interest

*NA* not available, *NMA* network meta-analysis

In risk of bias analyses, a majority of the studies included in the NMA were assessed at low or unclear risk (Supplementary Figure 1).

### Assessment of heterogeneity

Between-trial heterogeneity for 11 of the 40 pairwise meta-analyses of each treatment comparison for each endpoint of interest with direct evidence was substantial (*I*^*2*^ values >50%, mainly for between-treatment comparisons for pain-free at 2 hours and sustained pain-free at 24 hours). This heterogeneity for some comparisons within these endpoints were supported by p-values for the Q-statistic. The heterogeneity did not appear to be a result of differences in study design, as this was almost identical across studies, but may have been related to different placebo effects caused by different methods of recruitment, investigator training or non-measured variables; as noted, the populations of each study appeared to be similar Table 3. Bubble plots showing the treatment effect by placebo response for all outcomes investigated in the NMA are provided in Supplementary Figure 2. As treatment effect was associated with placebo response (decreasing across all treatments in line with placebo response increases) for the endpoints pain freedom at 2 hours and sustained pain freedom over 24 hours, models adjusted for baseline risk were used for these outcomes.

### Base case

#### Pain freedom at 2 hours

**Fig. 2:**
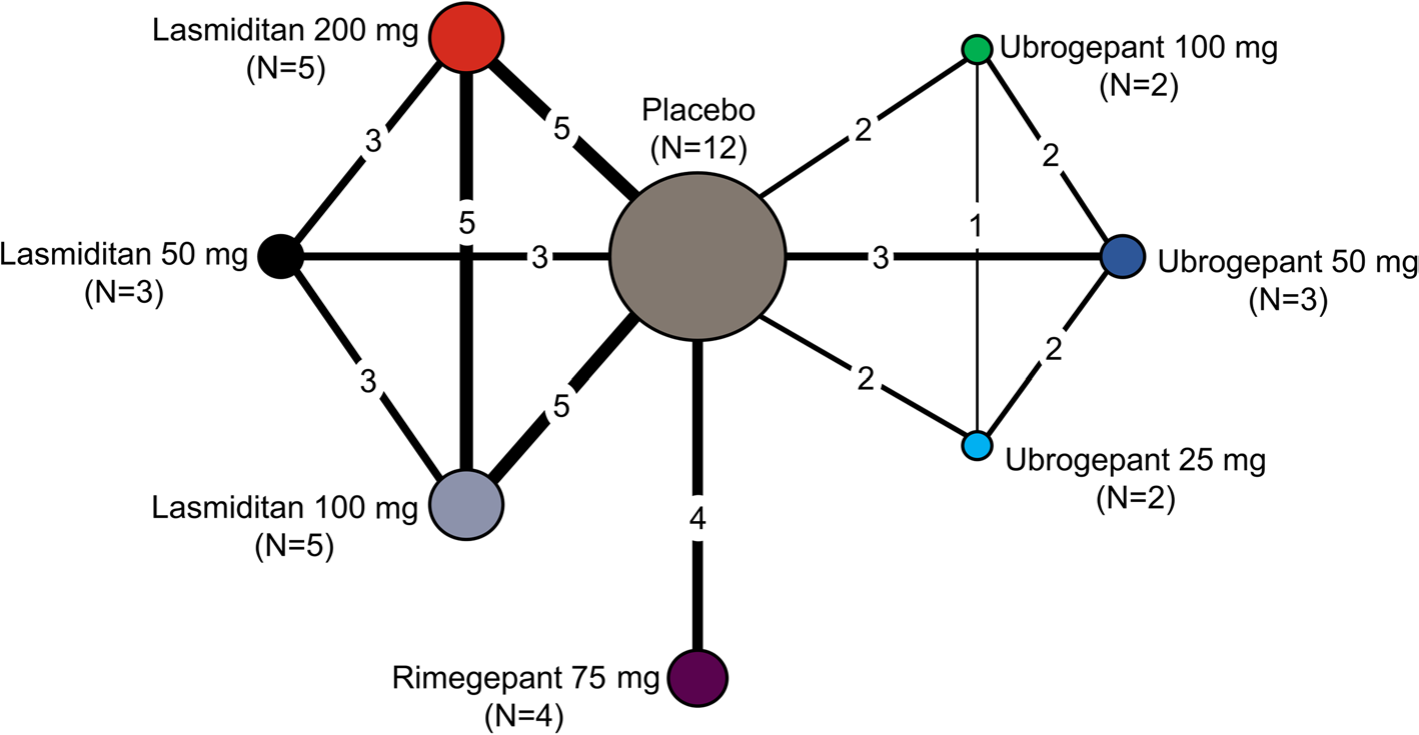
Network diagrams: base case analysis for pain freedom at 2 hours^a^ (12 randomised controlled trials). ^a^Assessed using Bayesian fixed-effects model adjusted for baseline risk (36 observations; residual deviance = 36.26). Lines are weighted according to the number of studies comparing the two treatments, and the radius of the circle indicates the number of studies within a given treatment arm

The base case network diagram for pain-freedom at 2 hours is shown in Fig. 2. In total, 12 studies and eight treatment nodes were included for this outcome Table 2.

**Fig. 3:**
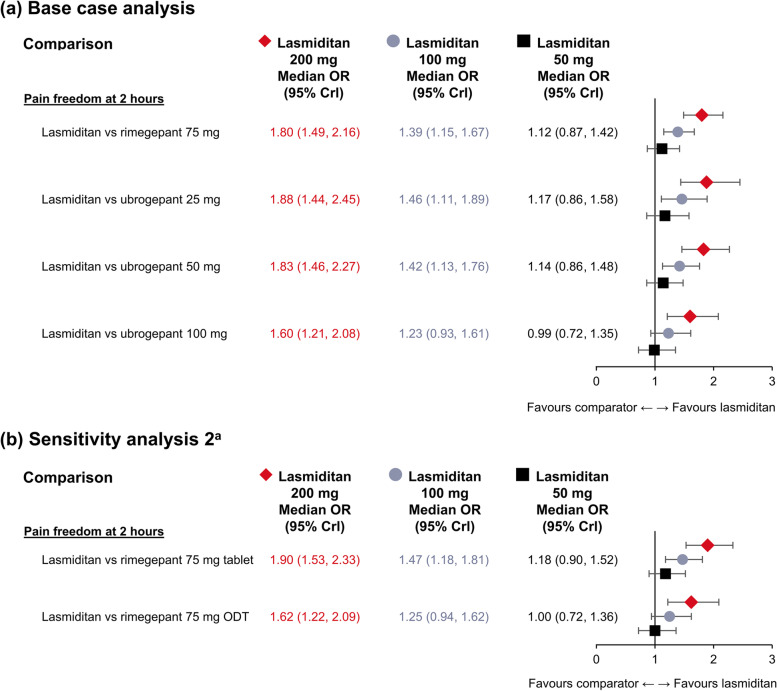
NMA results for pain freedom at 2 hours. ^a^Sensitivity analysis 2 analysed rimegepant according to its mode of administration (tablet or ODT). Pairwise treatment comparisons – results from Bayesian fixed-effects NMA adjusted for baseline risk (**base case analysis:** 36 observations, residual deviance = 36.26 [adjusted baseline risk: mean -0.54 (95% Crl -0.73, -0.28)]; **sensitivity analysis 2:** 36 observations, residual deviance = 35.74 [adjusted baseline risk: mean -0.52 (95% Crl -0.71, -0.25)]). Crl, credible interval; NMA, network meta-analysis; ODT, oral disintegrating tablet; OR, odds ratio

All doses of lasmiditan (50, 100, 200 mg) and both gepants exhibited statistically significant higher odds of inducing pain freedom at 2 hours versus placebo (Supplementary Figure 3). The odds of achieving pain freedom at 2 hours were statistically significantly higher with lasmiditan 200 mg than with all doses of both gepants, and with lasmiditan 100 mg than with rimegepant 75 mg and ubrogepant 25 mg and 50 mg (Fig. 3a). Lasmiditan 50 mg presented higher odds of inducing pain freedom at 2 hours than rimegepant 75 mg, and ubrogepant 25 and 50 mg; however, these differences were not significant. Using a fixed effects analysis with no adjustment for baseline risk showed a small but perceptible difference between the unadjusted and adjusted analyses (Supplementary Figure 4).

The results of both sensitivity analysis 1, including only phase III trials (shown in Supplementary Figure 5), and sensitivity analysis 2, which analysed rimegepant according to its mode of administration (tablet or ODT) (Fig. 3b), were consistent with those of the base case model.

#### Pain relief at 2 hours and 1 hour

A total of 12 studies and eight treatment nodes were included for the base case outcome of pain relief at 2 hours. In total, seven studies (data for ACHIEVE I and ACHIEVE II were extracted from a pooled analysis of both studies) and six treatment nodes were included for the base case outcome of pain relief at 1 hour Table 2.

All doses of lasmiditan and both gepants showed statistically significant higher odds of inducing a reduction in headache pain at both 2 hours and 1 hour versus placebo (Supplementary Figure 3). Lasmiditan (100 and 200 mg) was associated with statistically significant higher odds of achieving a reduction in headache pain at 2 hours and 1 hour versus both gepants (Supplementary Figure 6a, Supplementary Figure 7). The odds of achieving a reduction in headache pain with lasmiditan 50 mg were comparable to those with rimegepant 75 mg (at both 2 hours and 1 hour) and ubrogepant 25 (at 2 hours), 50 (at both 2 hours and 1 hour) and 100 mg (at 2 hours).

The results of both sensitivity analysis 1 (shown in Supplementary Figure 5), and sensitivity analysis 2 (Supplementary Figure 6b, pain relief at 2 hours only) were consistent with those of the base case models (note: sensitivity analysis 2 could not be performed for pain relief at 1 hour as no suitable data were available for rimegepant tablets. Exceptions included that although lasmiditan 100 and 200 mg improved the odds of achieving pain relief at 2 hours versus ubrogepant 100 mg (sensitivity 1) and rimegepant 75 mg ODT (sensitivity 2), these differences did not reach significance.

#### MBS freedom at 2 hours

In total, nine studies and eight treatment nodes were included for this outcome Table 2.

All doses of lasmiditan and of both gepants demonstrated statistically significant higher odds of inducing MBS freedom at 2 hours versus placebo (Supplementary Figure 3). Lasmiditan 200 mg was associated with higher odds of achieving MBS freedom at 2 hours compared with all doses of both rimegepant and ubrogepant, but the differences did not reach significance; little difference was seen between lasmiditan 100 mg and all doses of both rimegepant and ubrogepant (Supplementary Figure 8a). Lasmiditan 50 mg presented similar odds of inducing MBS freedom versus ubrogepant 25 mg and lower odds of inducing MBS freedom versus rimegepant 75 mg, and ubrogepant 50 and 100 mg (not statistically significant).

The pairwise results for sensitivity analyses 1 (Supplementary Figure 5) and 2 (Supplementary Figure 8b) for MBS free at 2 hours were generally consistent with those of the base case analysis.

#### Sustained pain freedom over 24 hours

In total, 11 studies and eight treatment nodes were included for this outcome Table 2.

All doses of lasmiditan and both gepants were associated with statistically significant higher odds of achieving sustained pain freedom over 24 hours versus placebo (Supplementary Figure 3). The odds of achieving sustained pain freedom with lasmiditan 200 mg were statistically significantly higher versus ubrogepant 25 mg and 50 mg and numerically (but not significantly) higher versus ubrogepant 100 mg and rimegepant 75 mg (Supplementary Figure 9a). The odds of achieving sustained pain freedom over 24 hours with lasmiditan 100 and 50 mg were higher versus ubrogepant 25 and 50 mg and similar or lower versus rimegepant 75 mg and ubrogepant 100 mg (no statistical significance). Using a fixed effects analysis with no adjustment for baseline risk showed a small but perceptible difference between the unadjusted and adjusted analyses (Supplementary Figure 10).

Pairwise results for sensitivity analyses 1 (Supplementary Figure 5) and 2 (Supplementary Figure 9b) for sustained pain freedom over 24 hours were consistent with those of the base case analysis.

### Median event rates and SUCRA values

Rankings and SUCRA percentages of the interventions by study and outcomes are presented in Supplementary Table 5. Lasmiditan 200 mg ranked highest on most of the base case outcomes analysed.

### Very early-onset outcomes (sensitivity 3 analyses)

In total, seven studies were included for the pain relief at 30 min outcome and six for other outcomes included in these analyses (pain freedom 1 hour and at 30 min and MBS freedom at 1 hour), covering seven and six treatment nodes, respectively (Table 2).

The odds of achieving pain freedom at 1 hour was statistically significantly higher with all doses of lasmiditan than ubrogepant 50 mg (Supplementary Figure 11). The odds of achieving pain freedom at 30 min with lasmiditan 200 mg were higher than with ubrogepant 50 mg, and lower with lasmiditan 100 mg and 50 mg versus ubrogepant 50 mg, but none of these differences met statistical significance (Supplementary Figure 11).

When pain relief at 30 min was considered, ORs were statistically significantly higher with lasmiditan 200 mg versus rimegepant 75 mg ODT and ubrogepant 50 mg, and with lasmiditan 100 mg versus ubrogepant 50 mg, but did not achieve statistical significance in the pairwise comparison of lasmiditan 100 mg versus rimegepant 75 mg ODT or any comparison involving lasmiditan 50 mg. The odds of achieving MBS freedom at 1 hour were statistically significantly higher with both lasmiditan 100 mg and 200 mg versus ubrogepant 50 mg but did not differ for lasmiditan 50 mg versus ubrogepant 50 mg (Supplementary Figure 11).

### ADRs

According to US prescribing information, the most common ADRs (with an incidence ≥2%, and higher than with placebo) reported in clinical trials with lasmiditan 50/100/200 mg were dizziness (9%/15%/17%), paraesthesia (3%/7%/9%), sedation (6%/6%/7%), fatigue (4%/5%/6%), nausea and/or vomiting (3%/4%/4%) and muscle weakness (1%/1%/2%) [41]. Nausea (2%) was the most common ADR reported with rimegepant ODT [42], and nausea (2%/4%), somnolence (sedation and fatigue) (2%/3%) and dry mouth (<1%/2%) were the most common ADRs reported with ubrogepant 50/100 mg [43].

## Discussion

In the current analyses, lasmiditan 200 mg showed statistically significant higher efficacy than all doses of both rimegepant and ubrogepant on the endpoints of pain freedom at 2 hours, pain relief at 2 hours and 1 hour, and numerically higher efficacy than both gepants for sustained pain freedom and freedom from MBS. Lasmiditan 100 mg showed statistically significant higher efficacy than both rimegepant 75 mg and ubrogepant 25 mg and 50 mg, and similar efficacy to ubrogepant 100 mg, on the endpoint of pain freedom at 2 hours. Additionally, lasmiditan 100 mg showed statistically significant higher efficacy than all doses of both rimegepant and ubrogepant on the endpoints of pain relief at 2 hours and 1 hour, with similar efficacy to ubrogepant 25 mg and 50 mg on the endpoint of sustained pain freedom and to ubrogepant 25 mg on the endpoint of MBS freedom. No statistically significant differences were found between lasmiditan 50 mg and ubrogepant or rimegepant for any outcome. Overall, the results of base case analyses were supported by those of sensitivity analyses. According to US labelling information, lasmiditan use is associated with mainly neurological events, such as dizziness, fatigue, paraesthesia and sedation [41], whereas rimegepant and ubrogepant are associated with low incidences of nausea (rimegepant) and somnolence, dry mouth and nausea (ubrogepant) [42, 43].

People with migraine consider rapid and sustained freedom from pain and MBS important attributes of an acute treatment for migraine, and these outcomes are recommended treatment goals for a migraine attack [4, 5, 44]. Insufficient efficacy or tolerability of an acute treatment for migraine can lead to non-adherence [45].

Our findings are supported by those of three other NMAs comparing the efficacy of lasmiditan, rimegepant and ubrogepant, although differences in design limit detailed comparisons between the NMAs. The NMA of Johnston et al. [28] included five phase III RCTs and compared the efficacy of lasmiditan, rimegepant (ODT only) and ubrogepant (at the same doses examined in the current NMA) in a subset of the outcomes included in the current study – pain freedom at 2 hours, pain relief at 2 hours, MBS freedom at 2 hours and sustained pain freedom over 24 hours. Johnston et al. [28] reported risk differences rather than ORs, precluding the comparison of risk data, but using SUCRA ranking, lasmiditan 200 mg was found to rank the highest of the investigated interventions on the outcomes of pain freedom at 2 hours, pain relief at 2 hours and MBS freedom at 2 hours, and was second to rimegepant ODT for sustained pain freedom over 24 hours. These findings agree closely with those of the current analysis, which also found lasmiditan 200 mg to rank highest on a majority of the outcomes assessed.

In another NMA, Agboola et al. [29] compared the efficacy of lasmiditan, rimegepant (ODT/tablet not differentiated), ubrogepant (using data from 10 RCTs) and two triptans, eletriptan and sumatriptan (23 RCTs). After adjusting for placebo response, the odds of achieving pain freedom (OR 1.43, 95% Crl 0.97, 2.06 vs rimegepant 75 mg) and pain relief (OR 1.16, 95% Crl 0.87, 1.52 vs rimegepant 75 mg and 1.15, 95% Crl 0.85, 1.58 vs ubrogepant 50/100 mg [pooled data]) at 2 hours were higher with lasmiditan (200/100 mg [pooled data]) [46]. Although in the same direction as the findings from the current NMA, these differences did not reach statistical significance. Additionally, the Crls reported in the current NMA are smaller than those reported in the NMA by Agboola et al. [29], and so provided greater precision around the estimate.

In the largest of these NMAs, Yang et al. [30] included a total of 64 double-blind RCTs with the aim of comparing the efficacy of lasmiditan, rimegepant, ubrogepant, triptans and other currently available migraine-specific acute treatments. In comparisons between lasmiditan (50 and 100 mg), rimegepant (75 mg ODT/tablet not differentiated) and ubrogepant (50 and 100 mg), no statistically significant differences in the odds of achieving pain freedom or pain relief at 2 hours were seen.

Notable differences between these NMAs and the current NMA include that CENTURION [17] and MONONOFU study findings [18] were unavailable at the time of publication of the above-mentioned NMAs; hence, none of the three NMAs included lasmiditan data from these sources. As CENTURION was a multicountry study and MONONOFU focused on an Asian population, we consider that inclusion of these studies in the current NMA has enriched the representativeness of its findings. Additionally, Yang et al. [30] did not include lasmiditan 200 mg in their analyses, limiting them to doses in widespread clinical use at the time the analysis was conducted. Johnston et al. [28] did not include phase II studies for any of the interventions studied, and the NMAs by Johnston et al. [28] and Yang et al. [30] used fixed-effect and random-effects models, respectively, with no adjustment for baseline risk (placebo response). In the current NMA, the assessment of heterogeneity identified different placebo responses across the included trials and, given the possible impact of this on treatment effects [37], the most affected models (e.g., those for the pain freedom outcomes) were adjusted for baseline risk when appropriate. Finally, none of these earlier NMAs examined the impact of lasmiditan, rimegepant or ubrogepant on the onset of pain outcomes prior to 2 hours. In the current NMA, lasmiditan 200 mg and 100 mg were associated with consistently statistically significant higher efficacy versus ubrogepant across a range of very early-onset pain outcomes, including pain freedom at 1 hour, MBS freedom at 1 hour and pain relief at 30 min.

Another difference between the current NMA and those by Johnson et al. [28], Yang et al. [30] and Agboola et al. [29] was that individual AEs were not included as an outcome in our NMA, for a number of reasons. First, published studies often report serious AEs or AEs only if they are above a particular threshold (e.g., 5% or 10%). Hence, if treatments have different AE profiles (as seen here for lasmiditan, rimegepant and ubrogepant), to what and how do you assign events to specific AEs when they are not reported? Assigning zeros into a network provides methodological challenges and requires additional assumptions to get convergence. A second problem involves the lack of a common comparator. The performance of NMAs and adjusted indirect comparisons is based on the assumption that the networks are connected by a common arm. In the current NMA all the included studies were placebo-controlled trials; hence, for the NMA to be valid, then all the study designs for the placebo arms needed to be similar. In our analyses of short-term efficacy outcomes (up to 2 hours) and the sustained pain freedom outcome (which excluded use of rescue medication [for a definition, see Table 1]) this assumption was valid. However, AEs are usually reported for the full trial period (generally 48 hours), and most of the included studies allowed the use of rescue therapy for those not responding to a first dose of treatment. As the timing and the type of rescue treatment allowed after the initial 2 hours differed between studies, and placebo recipients frequently required rescue medication, there was no longer a common placebo arm through which to join the active treatments in the NMA.

An attempt to assess safety by analysing discontinuations due to AEs as an outcome in the current NMA was precluded by a lack of such events across the included studies. We therefore summarised the reported ADRs for lasmiditan, rimegepant and ubrogepant, according to the US prescribing information. As expected, in light of their different mechanisms of action, the ADR profiles of the three agents differ notably. Lasmiditan use is associated with mainly neurological events (e.g., dizziness, fatigue, paraesthesia and sedation) [41], nausea is the most common ADR reported with rimegepant [42], and nausea and somnolence are the most common ADRs reported with ubrogepant [43]. These findings are in line with the NMA by Johnston et al. [28], which found dizziness, nausea and somnolence to be the most commonly reported AEs with lasmiditan, rimegepant and ubrogepant, respectively. Moreover, an analysis of the safety profile of lasmiditan using data from the SAMURAI and SPARTAN phase III RCTs (both included in the current NMA) found that lasmiditan use was associated with neurological treatment-emergent AEs, including dizziness, fatigue, paraesthesia and somnolence [47]. AEs associated with lasmiditan were mostly of short duration and mild or moderate in severity, occurring within ~30 to 50 min post-dosing [47]. Further published data characterising the tolerability profiles of rimegepant and ubrogepant are currently unavailable [48].

Of note, lasmiditan, rimegepant and ubrogepant all lack the vasoconstrictive effects associated with triptans, which have led to contraindications to triptan use in certain high-risk patients [8, 49].

This NMA used a connected network of studies that were well balanced in design and baseline characteristics. Nevertheless, there are a number of limitations to the study that should be recognised. When interpreting the results, it should be noted that due to issues with model convergence, random-effects models were not always feasible where there was evidence of heterogeneity. Steps taken to address heterogeneity in the models included considering the use of informative priors with no improvement or use of models adjusted for baseline risk for outcomes associated with substantial heterogeneity: pain freedom at 2 hours and sustained pain freedom over 24 hours. However, differences in the designs of the included studies relating to the administration of rescue medication and variations observed in the placebo response may have impacted the results. Although all the included studies belong to a narrow publication year range, 2012 to 2020, with designs consistent with then current clinical guidelines, many factors can influence the placebo effect, some of which may have played a role in the heterogeneity observed across the included studies (e.g., geographical distribution) [37]. Additionally, ‘placebo response reduction training’, used in the CENTURION study [17] to reduce patient and study staff expectations of therapeutic benefit, has also been shown to decrease the placebo effect [50]. These limitations were addressed in our study by applying appropriate methodological and statistical approaches; however, their potential influence still needs to be considered when interpreting the NMA results. This also applies to the robustness of the models.

Another limitation of our study is that the number of studies included for each outcome was relatively small, which can lead to instability especially when using random-effects models. Limited events hindered the precise estimates of safety outcomes, as well as a lack of long-term efficacy and safety data. Analyses could not be performed for discontinuation due to AEs (as a result of poor model fit). Hence, the safety profiles of each treatment could not be compared quantitatively as a consequence of their different mechanisms of action. This NMA therefore focused only on the efficacy of lasmiditan and the gepants rimegepant and ubrogepant. However, it is noteworthy that discontinuations due to AEs were very low with all treatments. Most of the included studies presented results for the treatment of a single migraine attack; hence, outcomes are uncertain when these drugs are used over time for repeated attacks. Finally, some data included in the models were estimated from published information. When percentages of patients with events were reported instead of absolute values, absolute values were estimated from the percentages. When data were reported in figures instead of tables or text, digitalisation of the figures was used to extract the information. Such approaches might have introduced slight variation (minimal impact at decimal places) from the true values.

## Conclusion

The results of this NMA indicate that lasmiditan 200 and 100 mg might be an appropriate acute treatment option for people with migraine, offering greater efficacy at 2 hours and a faster onset of action than both rimegepant and ubrogepant. Differently from rimegepant and ubrogepant, lasmiditan use is associated with mainly neurological events, which are mostly mild or moderate in severity and self-limiting.

## Supplementary Information

**Additional file 1.**

## Data Availability

The datasets used and/or analysed during the current study are available from the corresponding author on reasonable request.

## Abbreviations

5-HT_1F_: serotonin (5-hydroxytryptamine) IF receptor
AE: adverse event
ADR: adverse drug reaction
CGRP: calcitonin gene-related peptide
CrI: credible interval
CVD: cardiovascular disease
ECG: electrocardiogram
ER: emergency room
GI: gastrointestinal
HIV: human immunodeficiency virus
*I*^2^: inconsistency parameter
ITT: intention to treat
MBS: most bothersome symptom
MIDAS: Migraine Disability Assessment Test
NA: not available
NMA: network meta-analysis
NSAID: non-steroidal anti-inflammatory drug
ODT: oral disintegrating tablet
OR: odds ratio
PICOS: population, intervention, comparator and outcome selection
PRISMA: Preferred Reporting Items for Systematic Reviews and Meta-Analyses
RCT: randomised controlled trial
SLR: systematic literature review
SUCRA: surface under the cumulative ranking curve
SUD: substance use disorder
