# Supplementary material for: Relative efficacy of lasmiditan versus rimegepant and ubrogepant as acute treatments for migraine: network meta-analysis findings
Source: J Headache Pain. 2022 Jul 6;23(1):76. doi: 10.1186/s10194-022-01440-w (PMC9258126; doi:10.1186/s10194-022-01440-w)
Supplement: Supplementary file 1 — Additional file 1. [file 10194_2022_1440_MOESM1_ESM.docx]

# Supplementary material

**Supplementary Table 1** Search strategies

**Embase 1974 to 2018 April 03**

| **#** | | **Searches** | **Results** | |
| --- | --- | --- | --- | --- |
| 1 | | exp migraine/ | 55,777 | |
| 2 | | migrain$.ti,ab,kw . | 47,248 | |
| 3 | | 1 or 2 | 62,219 | |
| 4 | | Clinical trial/ | 967,992 | |
| 5 | | Controlled clinical trial/ | 458,812 | |
| 6 | | randomised controlled trial/ | 496,175 | |
| 7 | | Randomization/ | 77,559 | |
| 8 | | multicenter study/ | 181,397 | |
| 9 | | phase 3 clinical trial/ | 33,368 | |
| 10 | | phase 4 clinical trial/ | 2913 | |
| 11 | | Double Blind procedure/ | 148,403 | |
| 12 | | Single Blind procedure/ | 30,929 | |
| 13 | | crossover procedure/ | 54,983 | |
| 14 | | placebo/ | 322,972 | |
| 15 | | Randomi?ed controlled trial$.tw . | 178,468 | |
| 16 | | RCT.tw . | 27,953 | |
| 17 | | (random$ adj2 allocat$).tw . | 36,334 | |
| 18 | | Single blind$.tw . | 20,914 | |
| 19 | | Double blind$.tw . | 188,096 | |
| 20 | | ((treble or triple) adj blind$).tw . | 788 | |
| 21 | | placebo$.tw . | 271922 | |
| 22 | | prospective study/ | 437,826 | |
| 23 | | or/4-22 | 1,971,402 | |
| 24 | | case study/ | 53,276 | |
| 25 | | case report.tw . | 360,240 | |
| 26 | | abstract report/ or letter/ | 1,050,270 | |
| 27 | | conference abstract.pt. | 2,955,112 | |
| 28 | | Editorial.pt. | 561,572 | |
| 29 | | Letter.pt. | 1,010,462 | |
| 30 | | Note.pt. | 709,291 | |
| 31 | | or/24-30 | 5,645,262 | |
| 32 | | 23 not 31 | 1,530,873 | |
| 33 | | lasmiditan/ | 67 | |
| 34 | | (lasmiditan or COL-144 or UNII-760I9WM792 or AK118948 or LY- 573144).ti,ab,kw . | 42 | |
| 35 | | paracetamol/ | 80,476 | |
| 36 | | (acetaminophen or paracetamol or APAP or tylenol).ti,ab,kw . | 34,860 | |
| 37 | | acetylsalicylic acid/ | 194,208 | |
| 38 | | (Acetylsalicylic Acid or aspirin or ecotrin).ti,ab,kw . | 76,526 | |
| 39 | | celecoxib/ | 20,022 | |
| 40 | | (celecoxib or celebrex).ti,ab,kw . | 8059 | |
| 41 | | diclofenac/ | 36,298 | |
| 42 | | (diclofenac or volteran or volterol).ti,ab,kw . | 15,381 | |
| 43 | | Mefenamic acid/ | 5563 | |
| 44 | | Mefenamic acid.ti,ab,kw . | 1560 | |
| 45 | | flurbiprofen/ | 7291 | |
| 46 | | flurbiprofen.ti,ab,kw . | 2880 | |
| 47 | | ibuprofen/ | 45,034 | |
| 48 | | (ibuprofen or advil or motrin or proprinal).ti,ab,kw . | 16,813 | |
| 49 | | indometacin/ | 76,366 | |
| 50 | | (indomethacin or indocin or tivorbex).ti,ab,kw . | 41,068 | |
| 51 | | ketoprofen/ | 12,266 | |
| 52 | | ketoprofen.ti,ab,kw . | 4969 | |
| 53 | | ketorolac/ | 8578 | |
| 54 | | ketorolac.ti,ab,kw . | 3775 | |
| 55 | | naproxen/ | 24,628 | |
| 56 | | (naproxen or Aleve or Naprosyn).ti,ab,kw . | 7899 | |
| 57 | | nimesulide/ | 4464 | |
| 58 | | nimesulide.ti,ab,kw . | 2230 | |
| 59 | | piroxicam/ | 10,931 | |
| 60 | | (piroksikam or piroxi?am).ti,ab,kw . | 4044 | |
| 61 | | tolfenamic acid/ | 902 | |
| 62 | | (Tolfenamic Acid or clotam).ti,ab,kw . | 404 | |
| 63 | | phenazone/ | 6326 | |
| 64 | | (phenazon or antipyrine or analgesine).ti,ab,kw . | 3214 | |
| 65 | | etodolac/ | 2594 | |
| 66 | | (etodolac or lodine).ti,ab,kw . | 969 | |
| 67 | | zaltoprofen/ | 189 | |
| 68 | | zaltoprofen$.ti,ab,kw . | 109 | |
| 69 | | pranoprofen/ | 274 | |
| 70 | | pranoprofen.ti,ab,kw . | 143 | |
| 71 | | loxoprofen/ | 1095 | |
| 72 | | loxoprofen.ti,ab,kw . | 413 | |
| 73 | | lornoxicam/ | 1002 | |
| 74 | | lornoxicam.ti,ab,kw . | 637 | |
| 75 | | oxaprozin/ | 706 | |
| 76 | | oxaprozin.ti,ab,kw . | 217 | |
| 77 | | almotriptan/ | 1152 | |
| 78 | | (almotriptan or axert).ti,ab,kw . | 353 | |
| 79 | | eletriptan/ | 1327 | |
| 80 | | (eletriptan or relpax).ti,ab,kw . | 371 | |
| 81 | | frovatriptan/ | 936 | |
| 82 | | (Frovatriptan or frova).ti,ab,kw . | 337 | |
| 83 | | sumatriptan/ | 7750 | |
| 84 | | (sumatriptan or Imitrex or Imigran or Sumatran or Sumatriptanum or Sumax).ti,ab,kw . | 3910 | |
| 85 | | naratriptan/ | 1621 | |
| 86 | | (naratriptan or Amerge or Naramig).ti,ab,kw . | 399 | |
| 87 | | rizatriptan/ | 2251 | |
| 88 | | (rizatriptan or maxalt).ti,ab,kw . | 680 | |
| 89 | | zolmitriptan/ | 2696 | |
| 90 | | zolmitriptan.ti,ab, kw . | 755 | |
| 91 | | chlorpromazine/ | 44,525 | |
| 92 | | (Chlorpromazine or cpz orThorazine or Largactil).ti,ab,kw . | 13,899 | |
| 93 | | prochlorperazine/ | 5793 | |
| 94 | | Prochlorperazine.ti,ab,kw . | 970 | |
| 95 | | promethazine/ | 13,591 | |
| 96 | | promethazine.ti,ab,kw . | 2703 | |
| 97 | | propyphenazone/ | 900 | |
| 98 | | Propyphenazone.ti,ab,kw . | 234 | |
| 99 | | isometheptene/ | 403 | |
| 100 | | Isometheptene.ti,ab,kw . | 60 | |
| 101 | | lidocaine/ | 68,585 | |
| 102 | | (lidocaine or xylocaine or lignocaine).ti,ab,kw . | 30,693 | |
| 103 | | dipyrone/ | 7708 | |
| 104 | | (Metamizole or dipyrone).ti,ab,kw . | 1773 | |
| 105 | | magnesium sulfate/ | 15,044 | |
| 106 | | (magnesium sulphate or mgso4).ti,ab,kw . | 4987 | |
| 107 | | octreotide/ | 20,239 | |
| 108 | | (Octreotide or Sandostatin).ti,ab,kw . | 11,376 | |
| 109 | | valproic acid/ | 58,044 | |
| 110 | | (valproic acid or valproate).ti,ab,kw . | 23,354 | |
| 111 | | dexamethasone/ | 136,150 | |
| 112 | | dexamethasone.ti,ab,kw . | 69,405 | |
| 113 | | hydrocortisone/ | 122,485 | |
| 114 | | hydrocortisone.ti,ab,kw . | 19,731 | |
| 115 | | prednisone/ | 158,849 | |
| 116 | | prednisone.ti,ab,kw . | 41,402 | |
| 117 | | butorphanol/ | 2986 | |
| 118 | | Butorphanol.ti,ab,kw . | 1606 | |
| 119 | | codeine/ | 19,631 | |
| 120 | | Codeine.ti,ab,kw . | 6385 | |
| 121 | | pethidine/ | 22,574 | |
| 122 | | (Meperidine or pethidine).ti,ab,kw . | 6327 | |
| 123 | methadone/ | 29,626 |  |  |
| 124 | (methadone or Dolophine).ti,ab,kw . | 16,934 |  |  |
| 125 | tramadol/ | 17,795 |  |  |
| 126 | tramadol.ti,ab,kw . | 7128 |  |  |
| 127 | dihydroergotamine/ | 5105 |  |  |
| 128 | (Dihydroergotamine or DHE).ti,ab,kw . | 3676 |  |  |
| 129 | ergotamine/ | 5465 |  |  |
| 130 | Ergotamine.ti,ab,kw . | 1924 |  |  |
| 131 | domperidone/ | 8364 |  |  |
| 132 | (domperidone or Motilium).ti,ab,kw . | 3042 |  |  |
| 133 | droperidol/ | 9191 |  |  |
| 134 | Droperidol.ti,ab,kw . | 2685 |  |  |
| 135 | granisetron/ | 5102 |  |  |
| 136 | granisetron.ti,ab,kw . | 2159 |  |  |
| 137 | metoclopramide/ | 23,776 |  |  |
| 138 | Metochlopramide.ti,ab,kw . | 44 |  |  |
| 139 | zatosetron/ | 54 |  |  |
| 140 | zatosetron.ti,ab,kw . | 32 |  |  |
| 141 | butalbital/ | 1072 |  |  |
| 142 | Butalbital.ti,ab,kw . | 205 |  |  |
| 143 | ubrogepant/ | 25 |  |  |
| 144 | (ubrogepant or MK-1602 or UNII-AD0O8X2QJR or AD0O8X2QJR).ti,ab,kw . | 8 |  |  |
| 145 | rimegepant/ | 32 |  |  |
| 146 | (rimegepant or BMS-927711 or BMS 927711 or UNII-997WVV895X or CHEMBL2178422).ti,ab,kw . | 17 |  |  |
| 147 | Telcagepant/ | 278 |  |  |
| 148 | (Telcagepant or MK-0974 or "MK 0974" or MK0974 or UNII- D42O649ALL).ti,ab,kw . | 153 |  |  |
| 149 | or/33-148 | 1,087,233 |  |  |
| 150 | 3 and 32 and 149 | 4212 |  |  |
| 151 | limit 150 to (conference abstract or conference paper or "conference review " or editorial or letter or note) | 202 |  |  |
| 152 | 150 not 151 | 4010 |  |  |
| 153 | limit 152 to english language | 3646 |  |  |

**Ovid MEDLINE(R) Epub Ahead of Print, In-Process & Other Non-Indexed Citations, Ovid MEDLINE (R) Daily and Ovid MEDLINE (R) 1946 to Present**

| **#** | | **Searches** | | **Results** | |
| --- | --- | --- | --- | --- | --- |
| 1 | | exp Migraine Disorders/ | | 24,767 | |
| 2 | | migrain$.ti,ab,kw . | | 31,376 | |
| 3 | | or/1-2 | | 35,274 | |
| 4 | | Randomised Controlled Trials as Topic/ | | 115,057 | |
| 5 | | randomised controlled trial/ | | 456,829 | |
| 6 | | Random Allocation/ | | 93,649 | |
| 7 | | Double Blind Method/ | | 144,833 | |
| 8 | | Single Blind Method/ | | 24,861 | |
| 9 | | clinical trial/ | | 509,310 | |
| 10 | | clinical trial, phase i.pt. | | 17,899 | |
| 11 | | clinical trial, phase ii.pt. | | 28,901 | |
| 12 | | clinical trial, phase iii.pt. | | 13,549 | |
| 13 | | clinical trial, phase iv.pt. | | 1488 | |
| 14 | | controlled clinical trial.pt. | | 92,281 | |
| 15 | | randomised controlled trial.pt. | | 456,829 | |
| 16 | | multicenter study.pt. | | 230,691 | |
| 17 | | clinical trial.pt. | | 509,310 | |
| 18 | | exp Clinical Trials as topic/ | | 312,028 | |
| 19 | | (clinical adj trial$).tw . | | 303,418 | |
| 20 | | ((singl$ or doubl$ or treb$ or tripl$) adj (blind$3 or mask$3)).tw . | | 155,551 | |
| 21 | | PLACEBOS/ | | 33,839 | |
| 22 | | placebo$.tw . | | 193,782 | |
| 23 | | randomly allocated.tw . | | 23,924 | |
| 24 | | (allocated adj2 random$).tw . | | 26,944 | |
| 25 | | or/4-24 | | 1,445,995 | |
| 26 | | case report.tw . | | 268,289 | |
| 27 | | letter/ | | 982,254 | |
| 28 | | historical article/ | | 344,050 | |
| 29 | | 26 or 27 or 28 | | 1,580,483 | |
| 30 | | 25 not 29 | | 1,412,871 | |
| 31 | | (lasmiditan or COL-144 or UNII-760I9WM792 or AK118948 or LY-573144).ti,ab,kw . | | 31 | |
| 32 | | paracetamol/ | | 16,453 | |
| 33 | | (acetaminophen or paracetamol or APAP or tylenol).ti,ab,kw . | | 22,233 | |
| 34 | | acetylsalicylic acid/ | | 42,141 | |
| 35 | | (Acetylsalicylic Acid or aspirin or ecotrin).ti,ab,kw . | | 51,198 | |
| 36 | | celecoxib/ | | 3943 | |
| 37 | | (celecoxib or celebrex).ti,ab,kw . | | 5550 | |
| 38 | | diclofenac/ | | 7078 | |
| 39 | | (diclofenac or volteran or volterol).ti,ab,kw . | | 10,206 | |
| 40 | | Mefenamic acid/ | | 1009 | |
| 41 | | Mefenamic acid.ti,ab,kw . | | 1199 | |
| 42 | | flurbiprofen/ | | 1788 | |
| 43 | | flurbiprofen.ti,ab,kw . | | 2233 | |
| 44 | | ibuprofen/ | | 7915 | |
| 45 | | (ibuprofen or advil or motrin or proprinal).ti,ab,kw . | | 11,826 | |
| 46 | | indometacin/ | | 27,645 | |
| 47 | | (indomethacin or indocin or tivorbex).ti,ab,kw . | | 34,759 | |
| 48 | | ketoprofen/ | | 2566 | |
| 49 | | ketoprofen.ti,ab,kw . | | 3495 | |
| 50 | | ketorolac/ | | 1326 | |
| 51 | | ketorolac.ti,ab,kw . | | 2557 | |
| 52 | | naproxen/ | | 3813 | |
| 53 | | (naproxen or Aleve or Naprosyn).ti,ab,kw . | | 5588 | |
| 54 | | nimesulide.ti,ab,kw . | | 1523 | |
| 55 | | piroxicam/ | | 2685 | |
| 56 | | (piroksikam or piroxi?am).ti,ab,kw . | | 2841 | |
| 57 | | (Tolfenamic Acid or clotam).ti,ab,kw . | | 302 | |
| 58 | | phenazone/ | | 4433 | |
| 59 | | (phenazon or antipyrine or analgesine).ti,ab,kw . | | 2834 | |
| 60 | | etodolac/ | | 454 | |
| 61 | | (etodolac or lodine).ti,ab,kw . | | 632 | |
| 62 | | zaltoprofen$.ti,ab,kw . | | 56 | |
| 63 | | pranoprofen.ti,ab,kw . | | 89 | |
| 64 | | loxoprofen.ti,ab,kw . | | 257 | |
| 65 | | lornoxicam.ti,ab,kw . | | 342 | |
| 66 | | oxaprozin/ | | 0 | |
| 67 | | oxaprozin.ti,ab,kw . | | 144 | |
| 68 | | (almotriptan or axert).ti,ab,kw . | | 249 | |
| 69 | | (eletriptan or relpax).ti,ab,kw . | | 234 | |
| 70 | | (Frovatriptan or frova).ti,ab,kw . | | 215 | |
| 71 | | sumatriptan/ | | 2139 | |
| 72 | | (sumatriptan or Imitrex or Imigran or Sumatran or Sumatriptanum or Sumax).ti,ab,kw . | | 2872 | |
| 73 | | (naratriptan or Amerge or Naramig).ti,ab,kw . | | 284 | |
| 74 | | (rizatriptan or maxalt).ti,ab,kw . | | 433 | |
| 75 | | zolmitriptan.ti,ab,kw . | | 504 | |
| 76 | | chlorpromazine/ | | 17,123 | |
| 77 | | (Chlorpromazine or cpz orThorazine or Largactil).ti,ab,kw . | | 13,461 | |
| 78 | | prochlorperazine/ | | 1042 | |
| 79 | | Prochlorperazine.ti,ab,kw . | | 928 | |
| 80 | | promethazine/ | | 2969 | |
| 81 | | promethazine.ti,ab,kw . | | 2359 | |
| 82 | | Propyphenazone.ti,ab,kw . | | 162 | |
| 83 | | Isometheptene.ti,ab,kw . | | 37 | |
| 84 | | lidocaine/ | | 23,217 | |
| 85 | | (lidocaine or xylocaine or lignocaine).ti,ab,kw . | | 23,547 | |
| 86 | | dipyrone/ | | 1450 | |
| 87 | | (Metamizole or dipyrone).ti,ab,kw . | | 1213 | |
| 88 | | magnesium sulfate/ | | 4837 | |
| 89 | | (magnesium sulphate or mgso4).ti,ab,kw . | | 3236 | |
| 90 | | octreotide/ | | 7121 | |
| 91 | | (Octreotide or Sandostatin).ti,ab,kw . | | 7573 | |
| 92 | | valproic acid/ | | 11,616 | |
| 93 | | (valproic acid or valproate).ti,ab,kw . | | 15,555 | |
| 94 | | dexamethasone/ | | 48,417 | |
| 95 | | dexamethasone.ti,ab,kw . | | 52,149 | |
| 96 | | hydrocortisone/ | | 68,375 | |
| 97 | | hydrocortisone.ti,ab,kw . | | 16,888 | |
| 98 | | prednisone/ | | 37,580 | |
| 99 | | prednisone.ti,ab,kw . | | 25,691 | |
| 100 | | butorphanol/ | | 1027 | |
| 101 | | Butorphanol.ti,ab,kw . | | 1391 | |
| 102 | | codeine/ | | 4306 | |
| 103 | | Codeine.ti,ab,kw . | | 4780 | |
| 104 | | pethidine/ | | 5606 | |
| 105 | | (Meperidine or pethidine).ti,ab,kw . | | 5162 | |
| 106 | | methadone/ | | 11,484 | |
| 107 | | (methadone or Dolophine).ti,ab,kw . | | 12,478 | |
| 108 | | tramadol/ | | 2726 | |
| 109 | | tramadol.ti,ab,kw . | | 4235 | |
| 110 | | dihydroergotamine/ | | 1384 | |
| 111 | | (Dihydroergotamine or DHE).ti,ab,kw . | | 2563 | |
| 112 | | ergotamine/ | | 2290 | |
| 113 | | Ergotamine.ti,ab,kw . | | 1802 | |
| 114 | | domperidone/ | | 1691 | |
| 115 | | (domperidone or Motilium).ti,ab,kw . | | 2216 | |
| 116 | | droperidol/ | | 1966 | |
| 117 | | Droperidol.ti,ab,kw . | | 2037 | |
| 118 | | granisetron/ | | 1044 | |
| 119 | | granisetron.ti,ab,kw . | | 1483 | |
| 120 | | metoclopramide/ | | 4707 | |
| 121 | | Metochlopramide.ti,ab,kw . | | 29 | |
| 122 | | zatosetron.ti,ab,kw . | | 22 | |
| 123 | | Butalbital.ti,ab,kw . | | 118 | |
| 124 | | (ubrogepant or MK-1602 or UNII-AD0O8X2QJR or AD0O8X2QJR).ti,ab,kw . | | 5 | |
| 125 | | (rimegepant or BMS-927711 or BMS 927711 or UNII-997WVV895X or CHEMBL2178422).ti,ab,kw . | | 14 | |
| 126 | | (Telcagepant or MK-0974 or "MK 0974" or MK0974 or UNII- D42O649ALL).ti,ab,kw . | | 93 | |
| 127 | | or/31-124 | | 474,602 | |
| 128 | | 3 and 30 and 127 | | 1889 | |
| 129 | | limit 128 to (case reports or clinical conference or comment or congresses or editorial or interview or  letter or new s or new spaper article or patient education handout or periodical index or personal narratives or portraits or published erratum or "review " or "scientific integrity review ") | | 511 | |
| 130 | | 128 not 129 | | 1378 | |
| 131 | | limit 130 to english language | | 1282 | |

**Cochrane CENTRAL**

| **ID** | **Search** | **Hits** |
| --- | --- | --- |
| #2 | MeSH descriptor: [Migraine Disorders] explode all trees | 1959 |
| #3 | migraine:ti,ab,kw (Word variations have been searched) | 4360 |
| #4 | #2 or #3 | 4360 |
| #5 | lasmiditan:ti,ab,kw (Word variations have been searched) | 9 |
| #6 | acetaminophen:ti,ab,kw | 3695 |
| #7 | paracetamol:ti,ab,kw | 5596 |
| #8 | acetylsalicylic acid:ti,ab,kw | 7540 |
| #9 | aspirin:ti,ab,kw | 10,091 |
| #10 | celecoxib:ti,ab,kw | 1329 |
| #11 | diclofenac:ti,ab,kw | 4072 |
| #12 | Mefenamic acid:ti,ab,kw | 320 |
| #13 | flurbiprofen:ti,ab,kw | 764 |
| #14 | ibuprofen:ti,ab,kw | 3297 |
| #15 | indometacin:ti,ab,kw | 1053 |
| #16 | ketoprofen:ti,ab,kw | 1018 |
| #17 | ketorolac:ti,ab,kw | 1907 |
| #18 | naproxen:ti,ab,kw | 1853 |
| #19 | nimesulide:ti,ab,kw | 334 |
| #20 | piroxicam:ti,ab,kw | 1131 |
| #21 | Tolfenamic Acid:ti,ab,kw | 58 |
| #22 | phenazone:ti,ab,kw | 122 |
| #23 | etodolac:ti,ab,kw | 224 |
| #24 | zaltoprofen:ti,ab,kw | 17 |
| #25 | pranoprofen:ti,ab,kw | 30 |
| #26 | loxoprofen:ti,ab,kw | 105 |
| #27 | almotriptan:ti,ab,kw | 81 |
| #28 | eletriptan:ti,ab,kw | 67 |
| #29 | Frovatriptan:ti,ab,kw | 66 |
| #30 | sumatriptan:ti,ab,kw | 729 |
| #31 | naratriptan:ti,ab,kw | 66 |
| #32 | rizatriptan:ti,ab,kw | 154 |
| #33 | zolmitriptan:ti,ab,kw | 146 |
| #34 | chlorpromazine:ti,ab,kw | 1315 |
| #35 | prochlorperazine:ti,ab,kw | 360 |
| #36 | Propyphenazone:ti,ab,kw | 25 |
| #37 | Isometheptene:ti,ab,kw | 34 |
| #38 | lidocaine:ti,ab,kw | 8901 |
| #39 | dipyrone:ti,ab,kw | 615 |
| #40 | magnesium sulfate:ti,ab,kw | 1633 |
| #41 | octreotide:ti,ab,kw | 1221 |
| #42 | valproic acid:ti,ab,kw | 1651 |
| #43 | dexamethasone:ti,ab,kw | 7673 |
| #44 | hydrocortisone:ti,ab,kw | 8604 |
| #45 | prednisone:ti,ab,kw | 7606 |
| #46 | butorphanol:ti,ab,kw | 362 |
| #47 | Codeine:ti,ab,kw | 1412 |
| #48 | pethidine:ti,ab,kw | 2000 |
| #49 | methadone:ti,ab,kw | 2249 |
| #50 | tramadol:ti,ab,kw | 2569 |
| #51 | dihydroergotamine:ti,ab,kw | 400 |
| #52 | Ergotamine:ti,ab,kw | 196 |
| #53 | domperidone:ti,ab,kw | 607 |
| #54 | droperidol:ti,ab,kw | 1039 |
| #55 | granisetron:ti,ab,kw | 893 |
| #56 | metoclopramide:ti,ab,kw | 2638 |
| #57 | zatosetron:ti,ab,kw | 3 |
| #58 | Butalbital:ti,ab,kw | 27 |
| #59 | ubrogepant:ti,ab,kw | 2 |
| #60 | rimegepant:ti,ab,kw | 1 |
| #61 | telcagepant:ti,ab,kw | 31 |
| #62 | oxaprozin:ti,ab,kw | 58 |
| #63 | Promethazine:ti,ab,kw | 684 |
| #64 | #5 or #6 or #7 or #8 or #9 or #10 or #11 or #12 or #13 or #14 or #15 or #16 or #17 or #18 or #19 or #20 or #21 or #22 or #23 or #24 or #25 or #26 or #27 or #28 or #29 or #30 or #31 | 33,215 |
| #65 | #32 or #33 or #34 or #35 or #36 or #37 or #38 or #39 or #40 or #41 or #42 or #43 or #44 or #45 or #46 or #47 or #48 or #49 or #50 or #51 or #52 or #53 or #54 or #55 or #56 or #57 or #58 or #59 or #60 or #61 or #62 or #63 | 49,786 |
| #66 | #64 or #65 | 78,291 |
| #67 | #4 and #66 in Trials | 1502 |

**Supplementary Table 2** Eligibility criteria for the SLR

| Patient population | All patients diagnosed with moderate or severe migraine (with or without aura) per international guidelines  Subgroups of interest:   - Patients with cardiovascular comorbidities (including confirmed cardiovascular disease or cardiovascular risk factors) - Adult patients (≥18 years) - Adolescent patients (≥12 years) - Paediatric patients (≥6 years) - Menstrual-related migraine |
| --- | --- |
| Interventions/Comparators | Key interventions – any combinations in all routes of administrations  Lasmiditan  Ubrogepant  Rimegepant  NSAIDs   - Aspirin - Celecoxib - Ibuprofen - Indomethacin - Naproxen - Nimesulide - Diclofenac - Tolfenamic acid - Ketorolac - Ketoprofen - Flurbiprofen - Mefenamic acid - Phenazone - Piroxicam   Triptans   - Sumatriptan - Zolmitriptan - Naratriptan - Rizatriptan - Almotriptan - Eletriptan - Frovatriptan   Ergots   - Ergotamine - Dihydroergotamine   Corticosteroids   - Dexamethasone - Hydrocortisone - Prednisone   Opioids   - Butorphanol - Codeine - Meperidine - Methadone - Tramadol   Others   - Acetaminophen - Butalbital - Propyphenazone - Isometheptene - Metamizole (dipyrone) - Lidocaine - Octreotide - Magnesium Sulphate (MgSO4) - Valproic acid/valproate |
| Outcomes | Efficacy endpoints (if studies include multiple migraine episodes, only first attack efficacy endpoints were extracted):   - Pain-free at 2 hours, without the use of rescue medication - Reduction in headache pain (‘headache relief’) at 1 and 2 hours (pain reduced from moderate or severe to none or mild without the use of rescue medication) - Sustained pain-free over 24 hours (pain-free within 2 hours, and pain-free at 24 hours with no use of rescue medication or recurrence within 24 hours) - Sustained pain relief over 24 hours (headache relief at 2 hours, sustained for 24 hours, with no use of rescue medication or a second dose of study medication) - Most bothersome symptom (MBS) free at 2 hours - Headache recurrence and use of rescue medications (e.g., second dose of study drug or other medication) with 2-48 hours   Safety endpoints (number and proportion experiencing event):   - Patients with any chest AEs (at least one chest symptom, defined as chest pressure, chest pain, radiating pain in arm, any other chest feelings, heavy arms, shortness of breath, palpitations and anxiety) - Patients with chest pressure - Patients with dizziness - Patients with paraesthesia - Patients with vertigo |
| Study design | - Phase II/III/IV RCTs - Crossover studies accepted if there was adequate washout (≥48 hours) between treatments - Relevant published systematic reviews - English language studies only - Non-RCT studies excluded |

AE, adverse event; NSAID, non-steroidal anti-inflammatory drug; RCT, randomised controlled trial, SLR, systematic literature review

Supplementary Table 3 Summary DIC and model choice for all outcomes (Base Case)

| **Endpoint** | **Model** | **DIC** | **Residual Deviance** |  | $\boldsymbol{p}_{\boldsymbol{D}}$ | **Sigma** $\boldsymbol{(\sigma}_{\boldsymbol{\delta}}\boldsymbol{)}$ | **Model Choice** |
| --- | --- | --- | --- | --- | --- | --- | --- |
| Pain Freedom at 2 hr  (36 data points) | FE | 59.13 | 40.12 |  | 19.01 | - | **FEadj** |
|  | **FEadj** | **56.55** | **36.26** |  | **20.3** | **-** |  |
|  | RE* | 58.96 | 34.69 |  | 24.27 | 0.13(0.01,0.3) |  |
|  | REadj | 57.77 | 34.57 |  | 23.2 | 0.07(0.0,0.2) |  |
|  | REip* | 58.76 | 36.19 |  | 22.57 | 0.09(0.01,0.24) |  |
| Pain relief at 1 hr (16 data points) | **FE** | **30.17** | **21.16** |  | **9.01** | **-** | **FE** |
|  | FEadj | 32.01 | 22.02 |  | 9.99 | - |  |
|  | RE* | 30.2 | 17.35 |  | 12.85 | 0.16 (0.01,0.43) |  |
|  | REadj | 31.24 | 17.34 |  | 13.9 | 0.2 (0.01,0.53) |  |
|  | REip* | 30.02 | 18.52 |  | 11.49 | 0.1 (0.01,0.3) |  |
| Pain relief at 2 hr  (36 data points) | **FE** | **56.3** | **37.31** |  | **18.99** | **-** | **FE** |
|  | FEadj | 57.62 | 37.55 |  | 20.07 | **-** |  |
|  | RE* | 57.51 | 35.03 |  | 22.48 | 0.08(0.0,0.21) |  |
|  | REadj | 59.22 | 35.39 |  | 23.83 | 0.0(0.08,0.22) |  |
|  | Reip* | 56.93 | 35.65 |  | 21.28 | 0.06(0.05,0.17) |  |
| Sustained pain free 2-24 hr (32 data points) | FE | 59.46 | 41.41 |  | 18.04 | - | **RE adj** |
|  | FEadj | 57.86 | 38.36 |  | 19.5 | - |  |
|  | RE* | 57.61 | 33.4 |  | 24. 21 | 0.20(0.02,0.43) |  |
|  | **RE adj** | **58.15** | **33.92** |  | **24.23** | **0.12(0.01,0.31)** |  |
|  | Reip* | 58.13 | 35.86 |  | 22.27 | 0.13(0.01,0.34) |  |
| MBS freedom at 2 hours  (26 data points) | **FE** | **35.08** | **19.07** |  | **16** | **-** | **FE** |
|  | FE adj | 37.14 | 20.22 |  | 16.92 | - |  |
|  | RE* | 37.05 | 19.61 |  | 17.44 | 0.05(0.0,0.17) |  |
|  | RE adj | 39.16 | 20.7 |  | 18.47 | 0.06(0.0,0.18) |  |
|  | REip* | 36.38 | 19.4 |  | 16.97 | 0.04(0.0,0.13) |  |

hr: hour; FE: Fixed Effects; RE: Random Effects; adj.: adjusted for baseline risk; RE*: Random Effects increased sample; REip: Random Effects informative Prior; DIC: Deviance Information Criteria; $p_{D}$: effective number of parameters; Sigma$:$between studies standard deviation

**Supplementary Table 4** Characteristics of studies included in the base case analysis

| **Study acronym and identifier** | **Design and duration of follow up** | **Interventions and dosing procedures** | **Main inclusion and exclusion criteria** |
| --- | --- | --- | --- |
| LASMIDITAN | | | |
| SAMURAI  Kuca et al. 2018 [1]  (NCT02439320) | Randomised, double-blind, placebo-controlled, multicentre, phase III, single attack study; follow-up visit 7 days after treated migraine attack | Lasmiditan (100 mg or 200 mg) vs placebo – study medication to be taken within 4-hours of migraine onset (moderate to severe pain); second dose for rescue allowed 2–24 hours after first dose | Inclusion: Adults ≥18 years; ≥1-year history of disabling migraine attacks with or without aura; onset before age 50; 3–8 migraine attacks/month (<15 headache days/month)  Exclusion: History of chronic migraine or other forms of primary or secondary headache disorder in past 12 months; ≥15 headache days/month within past 12 months; initiation of or change in migraine preventive medication within 3 months; known coronary artery disease; clinically significant arrythmia; uncontrolled hypertension; condition increasing risk of seizures |
| SPARTAN  Goadsby et al. 2019 [2]  (NCT02605174) | Prospective, randomised, double-blind, placebo-controlled, multicentre, phase III, single attack study; follow-up visit 7 days after treated migraine attack | Lasmiditan (50 mg, 100 mg, or 200 mg) vs placebo – study medication to be taken within 4-hours of migraine onset (moderate to severe pain); second dose for rescue or recurrence allowed 2–24 hours after first dose | Inclusion: Adults ≥18 years; ≥1-year history of disabling migraine attacks with or without aura; MIDAS score ≥11; onset before age 50; 3–8 migraine attacks/month (<15 headache days/month).  Exclusion: History of chronic migraine; other forms of primary or secondary headache disorder; ≥15 headache days/month within past 12 months; condition increasing risk of seizures; recurrent dizziness or vertigo; diabetes mellitus with complications; orthostatic hypotension with syncope; renal or hepatic impairment; current SUD past 3 years; imminent risk of suicide or suicide attempt within past 6 months |
| CENTURION  Ashina et al. 2021 [3]  (NCT03670810) | Randomised, double-blind, placebo-controlled, parallel assignment, multicentre, phase III, study; consistency over four migraine attacks | Lasmiditan (100 mg and 200 mg) vs placebo – study medication to be taken within 4-hours of migraine onset (moderate to severe pain) | Inclusion: ≥18 years; migraine with or without aura; history of disabling migraine for at least 1 year; migraine onset before the age of 50 years; 3–8 migraine attacks/month (<15 headache days/month) during the past 3 months; MIDAS score ≥11  Exclusion: Known hypersensitivity to lasmiditan; history of haemorrhagic stroke, epilepsy, or any other condition placing the participant at increased risk of seizures; history of recurrent dizziness and/or vertigo; history of diabetes mellitus with complications; history of orthostatic hypotension with syncope; significant renal or hepatic impairment; participants who are deemed to be at significant risk for suicide; history of chronic migraine or other forms of primary or secondary chronic headache disorder within past 12 months; use of more than 3 doses/month of either opioids or barbiturates; initiation of or a change in concomitant medication to reduce the frequency of migraine episodes within 3 months prior to screening; SUD within 1 year prior to screening; currently enrolled in any other clinical study involving an investigational product |
| MONONOFU  Sakai et al. 2021 [4]  (NCT03962738) | Randomised, double-blind, placebo-controlled, parallel assignment, multicentre, phase II, single attack study in a Japanese population; follow-up visit within 3 to 28 days of treated migraine attack | Lasmiditan (50 mg, 100 mg, or 200 mg) vs placebo – study medication to be taken within 4 hours of migraine onset (moderate to severe pain); unexcluded patient´s medication for rescue or recurrence allowed 2–24 hours after first dose | Inclusion: ≥18 years; migraine with or without aura; history of disabling migraine for at least 1 year; MIDAS score ≥11; migraine onset before the age of 50 years; 3–8 migraine attacks/month and <15 headache days/month during the past 3 months  Exclusion: Known hypersensitivity to lasmiditan; history of haemorrhagic stroke, epilepsy, or any other condition placing the patient at increased risk of seizures; history of recurrent dizziness and/or vertigo; history of diabetes mellitus with complications; history of orthostatic hypotension with syncope |
| Färkkilä et al. 2012  [5]  (NCT00883051) | Randomised, double-blind, placebo-controlled, parallel-group, multicentre, dose-ranging study, phase II, single attack; follow-up visit within 14 days of treated migraine attack | Lasmiditan (50 mg, 100 mg, 200, or 400mg) vs placebo – study medication to be taken within 4 hours of migraine onset (moderate to severe pain); second dose for rescue allowed (excl. triptans or ergotamines) 2 hours after first dose | Inclusion: Adults ≥18 years; ≥1-year history of acute migraine attacks with or without aura; onset before age 50; 1–8 migraine attacks/month  Exclusion: Use of migraine prophylaxis (unless discontinued at least 15 days prior to screening), vasoactive drugs, serotonin reuptake inhibitors, or known cytochrome P450 inhibitors |
| RIMEGEPANT | | | |
| Study 301  Lipton et al. 2018 [6]  (NCT03235479) | Randomised, double-blind, placebo-controlled, multicentre, phase III, single attack study; follow-up visit within 7 days of treated migraine attack | Rimegepant tablets (75 mg) vs placebo; rescue medication was allowed within 24 hours | Inclusion: Adults ≥18 years of age; ≥1-year history of migraine; 2–8 migraine attacks/month (moderate to severe intensity); <15 headache days/month within the past 3 months; patients receiving preventive migraine medications had to be receiving stable dose for at least 3 months before trial entry  Exclusion: History of any clinically significant or unstable medical condition, including alcohol or drug abuse and SUD; use of any biological investigational agents within 90 days of baseline visit; received non-biological investigational agents within 30 days before baseline visit |
| Study 302  Lipton et al. 2019 [7]  (NCT03237845) | Randomised, double-blind, placebo-controlled, multicentre, phase III, single attack study; follow-up visit within 7 days of treated migraine attack | Rimegepant tablets (75 mg) vs placebo – study medication to be taken when migraine of moderate to severe intensity occurred; use of second dose as rescue medication was allowed within 24 hours | Inclusion: Adults ≥18 years of age; ≥1-year history of migraine with or without aura; onset before age 50; 2–8 migraine attacks/month (moderate to severe intensity); <15 days/month with headache within the past 3 months; patients receiving preventive migraine medications had to be receiving stable dose for at least 3 months before trial entry  Exclusion: History of any clinically significant or unstable medical condition, including alcohol or drug abuse and SUD; use of any biological investigational agents within 90 days of baseline visit; received non-biological investigational agents within 30 days before baseline visit |
| Study 303  Croop et al. 2019 [8]  (NCT03461757) | Randomised, double-blind, placebo-controlled, multicentre, phase III, single attack study; follow-up visit within 7 days of treated migraine attack | Rimegepant ODT (75 mg) vs placebo – study medication to be taken when migraine attack of moderate to severe intensity occurred; rescue medications (e.g., aspirin, ibuprofen, acetaminophen [up to 1000 mg/day], naproxen [or any other NSAIDs], antiemetics, or baclofen) after 2 hours post-dose | Inclusion: Adults ≥18 years of age; ≥1-year history of migraine with or without aura; onset before age 50; 2–8 migraine attacks/month (moderate to severe intensity); <15 days per month with headache within the past 3 months  Exclusion: SUD within past 12 months; history of drug or other allergy that made them unsuitable for participation; ECG or laboratory test findings that raised safety or tolerability concerns |
| Marcus et al. 2014 [9] | Randomised, double-blind, multicentre, placebo-controlled, phase II, single attack study; follow-up visit within 7 days of treated migraine attack | Rimegepant tablets (10 mg, 25 mg, 75 mg, 150 mg, 300 mg, or 600 mg) vs sumatriptan (100 mg) and placebo – study medication to be taken at onset of moderate to severe migraine; use of rescue medication (aspirin, ibuprofen, acetaminophen, NSAID, antiemetics or baclofen) after 2 hours post-dose | Inclusion: Adults aged 18-65 years; ≥1-year history of migraine with or without aura; onset before age 50; duration of migraine attack 4–72 hours if untreated; 2–7 attacks/month (moderate to severe)  Exclusion: History of stroke/transient ischaemic attacks, ischaemic heart disease, coronary artery vasospasm, other significant underlying CVD, uncontrolled hypertension or diabetes, HIV; current diagnosis of major depression, other pain syndromes, psychiatric conditions, dementia, or significant neurological disorders, other than migraine; SUD within the past 12 months; use of known cytochrome P450 inhibitors or medications that may alter the pH of the stomach; use of barbiturates, opioids, triptans, ergotamines and muscle relaxants from within 2 days before randomisation and during the study |
| UBROGEPANT | | | |
| ACHIEVE I  Dodick et al. 2019 [10]  (NCT02828020) | Randomised, double-blind, placebo-controlled, multicentre, phase III, single attack study; follow-up visit within 7 days of treated migraine attack | Ubrogepant (50 or 100 mg) vs placebo; second dose or rescue medication allowed in patients with inadequate response or headache recurrence | Inclusion: Adults 18-75 years old; ≥1-year history of migraine attacks with or without aura; onset before age 50; duration of migraine attack 4–72 hours and separated by ≥48 hours; 2–8 migraine attacks/month with moderate to severe headache pain in previous 3 months  Exclusion: Taken medication for acute treatment of headache on ≥10 days/month in previous 3 months; history of aura with diplopia or impairment of level of consciousness, hemiplegic or retinal migraine; current diagnosis of new persistent daily headache, trigeminal autonomic cephalalgia, or painful cranial neuropathy; required hospital treatment of a migraine attack ≥3 times in previous 6 months; chronic non-headache pain condition requiring daily pain medication; history of malignancy in the prior 5 years; history of any prior GI conditions that may affect the absorption or metabolism; history of hepatitis within previous 6 months |
| ACHIEVE II  Lipton et al. 2019 [11] (NCT02867709) | Randomised, double-blind, placebo-controlled, multicentre, phase III, single attack study; follow-up visit within 7 days of treated migraine attack | Ubrogepant (25 mg, 50 mg, or 100 mg) vs placebo; second dose or rescue medication allowed in patients with inadequate response or headache recurrence | Inclusion: Adults 18–75 years old; ≥1-year history of migraine attacks with or without aura; onset before age 50; duration of migraine attack 4–72 hours and separated by ≥48 hours; 2–8 migraine attacks/month (moderate to severe intensity) in previous 3 months  Exclusion: Taken medication for acute treatment of headache on ≥10 days/month in the previous 3 months; history of migraine aura with diplopia or impairment of level of consciousness, hemiplegic or retinal migraine; current diagnosis of new persistent daily headache, trigeminal autonomic cephalalgia, or painful cranial neuropathy; required hospital treatment of a migraine attack ≥3 times in previous 6 months; chronic non-headache pain condition requiring daily pain medication; history of malignancy in the prior 5 years; history of any prior GI conditions that may affect the absorption or metabolism; history of hepatitis within previous 6 months |
| Voss et al. 2016 [12] | Randomised, double-blind, placebo-controlled, phase IIb, single attack study; follow-up visit 5 days post-treatment | Ubrogepant (1 mg, 10 mg, 25 mg, 50 mg, or 100 mg) vs placebo – study drug to be taken to treat a migraine of moderate to severe intensity; non-study medication allowed as rescue or recurrence treatment | Inclusion: Adults ≥18 years; ≥1-year history of acute migraine attacks with or without aura; onset before age 50; 1–8 migraine attacks/month  Exclusion: Difficulty distinguishing migraine attacks from tension type headaches; uncontrolled hypertension; basilar-type or hemiplegic migraine headache; >15 headache days/month or had taken medication for acute headache on >10 days/month in the 3 months prior to screening; acute attack within past 2 months that required inpatient or ER treatment; use of an opioid or barbiturate for migraine in the past 2 months; recent change in dose of migraine-prophylactic medication |

CVD, cardiovascular disease; ECG, electrocardiogram; ER, emergency room; GI, gastrointestinal; HIV, human immunodeficiency virus; MIDAS, Migraine Disability Assessment Test; NSAID, non-steroidal anti-inflammatory drug; ODT, oral disintegrating tablet; SUD, substance use disorder

**Supplementary Table 5** Median event rates and SUCRA values: base case analyses

|  | **Pain freedom at 2 hours** | | **Pain relief at 2 hours** | | **Pain relief at 1 hour** | | **MBS freedom at 2 hours** | | **Sustained pain freedom over 24 hours** | |
| --- | --- | --- | --- | --- | --- | --- | --- | --- | --- | --- |
| Treatment | Median event rate^a^, % (95% Crl) | SUCRA | Median event rate^a^, % (95% Crl) | SUCRA | Median event rate^a^, % (95% Crl) | SUCRA | Median event rate^a^,  % (95% Crl) | SUCRA | Median event rate^a^, % (95% Crl) | SUCRA |
| Lasmiditan 200 mg | **33.6 (30.2, 36.9)** | **100%** | 65.4 (62.0, 68.6) | 90% | **49.3 (45.4, 53.2)** | **99%** | **40.9 (37.0, 44.9)** | **84%** | **19.4 (9.9, 34.4)** | **97%** |
| Lasmiditan 100 mg | 28.1 (25.0, 31.2) | 84% | **66.1 (62.8, 69.3)** | **95%** | 46.7 (42.9, 50.6) | 81% | 39.5 (35.7, 43.4) | 68% | 15.6 (7.8, 28.7) | 63% |
| Lasmiditan 50 mg | 23.9 (20.1, 27.9) | 57% | 58.1 (53.4, 62.6) | 40% | 38.0 (33.1, 43.0) | 37% | 35.3 (30.4, 40.4) | 28% | 14.4 (6.8, 27.4) | 47% |
| Rimegepant 75 mg | 21.9 (19.4, 24.7) | 36% | 59.8 (56.4, 63.2) | 56% | 38.6 (33.1, 44.4)^b^ | 41% | 38.6 (34.9, 42.4) | 58% | 15.4 (7.6, 28.6) | 59% |
| Ubrogepant 100 mg | 24.1 (19.8, 28.9) | 58% | 58.8 (53.1, 64.3) | 47% | NA | NA | 38.9 (32.7, 45.4) | 61% | 16.6 (8.0, 31.2) | 73% |
| Ubrogepant 50 mg | 21.6 (18.5, 25.1) | 34% | 58.4 (53.8, 62.8) | 44% | 38.6 (33.9, 43.6) | 42% | 39.9 (35.0, 45.1) | 72% | 13.4 (6.5, 25.6) | 34% |
| Ubrogepant 25 mg | 21.2 (17.3, 25.6) | 30% | 56.3 (50.5, 61.9) | 27% | NA | NA | 35.2 (29.2, 41.7) | 30% | 12.6 (5.9, 24.7) | 27% |
| Placebo | 13.5 (12.6, 14.5) | 0% | 45.3 (43.9, 46.7) | 0% | 32.9 (31.4, 34.5) | 10% | 28.5 (27.2, 29.9) | 0% | 7.7 (3.7, 15.0) | 0% |

^a^Median event rate derived from posterior distributions

^b^Evaluated for orally disintegrating tablet only

SUCRA values range from 0% to 100%, where 100% reflects the best treatment with no uncertainty and 0% reflects the worst treatment with no uncertainty; higher value has the greatest probability of being the best

CrI, credible interval; NA, not available; SUCRA, surface under the cumulative ranking curve

## References

Kuca B, Silberstein SD, Wietecha L, Berg PH, Dozier G, Lipton RB; COL MIG-301 Study Group (2018) Lasmiditan is an effective acute treatment for migraine: a phase 3 randomized study. Neurology 91(24):e2222-e2232.

Goadsby PJ, Wietecha LA, Dennehy EB, Kuca B, Case MG, Aurora SK, Gaul C (2019) Phase 3 randomized, placebo-controlled, double-blind study of lasmiditan for acute treatment of migraine. Brain 142(7):1894-1904.

Ashina M, Reuter U, Smith T, Krikke-Workel J, Klise SR, Bragg S, Doty EG, Dowsett SA, Lin Q, Krege JH (2021) Randomized, controlled trial of lasmiditan over four migraine attacks: Findings from the CENTURION study. Cephalalgia 41(3):294-304.

Sakai F, Takeshima T, Homma G, Tanji Y, Katagiri H, Komori M (2021) Phase 2 randomized placebo-controlled study of lasmiditan for the acute treatment of migraine in Japanese patients. Headache 61(5):755-765.

Färkkilä M, Diener HC, Géraud G, Láinez M, Schoenen J, Harner N, Pilgrim A, Reuter U; COL MIG-202 study group (2012) Efficacy and tolerability of lasmiditan, an oral 5-HT(1F) receptor agonist, for the acute treatment of migraine: a phase 2 randomised, placebo-controlled, parallel-group, dose-ranging study. Lancet Neurol 11(5):405-413.

Lipton RB, Conway CM, Stock EG, Stock D, Morris BA, McCormack TJ, Frost M, Gentile K, Dubowchik GM, Coric V, Croop R (2018) Efficacy, safety, and tolerability of rimegepant 75 mg, an oral CGRP receptor antagonist, for the treatment of migraine: results from a phase 3, double blind, randomized, placebo-controlled trial, Study 301. Presented at: the 60^th^ Annual Scientific Meeting of the American Headache Society; June 28-July 1, 2018; San Francisco, CA, USA, Abstract 492562.

Lipton RB, Croop R, Stock EG, Stock DA, Morris BA, Frost M, Dubowchik GM, Conway CM, Coric V, Goadsby PJ (2019) Rimegepant, an oral calcitonin gene-related peptide receptor antagonist, for migraine. N Engl J Med 381(2):142-149.

Croop R, Goadsby PJ, Stock DA, Conway CM, Forshaw M, Stock EG, Coric V, Lipton RB (2019) Efficacy, safety, and tolerability of rimegepant orally disintegrating tablet for the acute treatment of migraine: a randomised, phase 3, double-blind, placebo-controlled trial. Lancet 394(10200):737-745.

Marcus R, Goadsby PJ, Dodick D, Stock D, Manos G, Fischer TZ (2014) BMS-927711 for the acute treatment of migraine: a double-blind, randomized, placebo controlled, dose-ranging trial. Cephalalgia 34(2):114-125.

Dodick DW, Lipton RB, Ailani J, Lu K, Finnegan M, Trugman JM, Szegedi A (2019) Ubrogepant for the treatment of migraine. N Engl J Med 381(23):2230-2241.

Lipton RB, Dodick DW, Ailani J, et al (2019) Effect of ubrogepant vs placebo on pain and the most bothersome associated symptom in the acute treatment of migraine: the ACHIEVE II randomized clinical trial. JAMA 322(19):1887-1898.

Voss T, Lipton RB, Dodick DW, Dupre N, Ge JY, Bachman R, Assaid C, Aurora SK, Michelson D (2016) A phase IIb randomized, double-blind, placebo-controlled trial of ubrogepant for the acute treatment of migraine. Cephalalgia 36(9):887-898.

**Supplementary Figure 1.** Risk of bias assessment for 11 studies* included in the NMA.
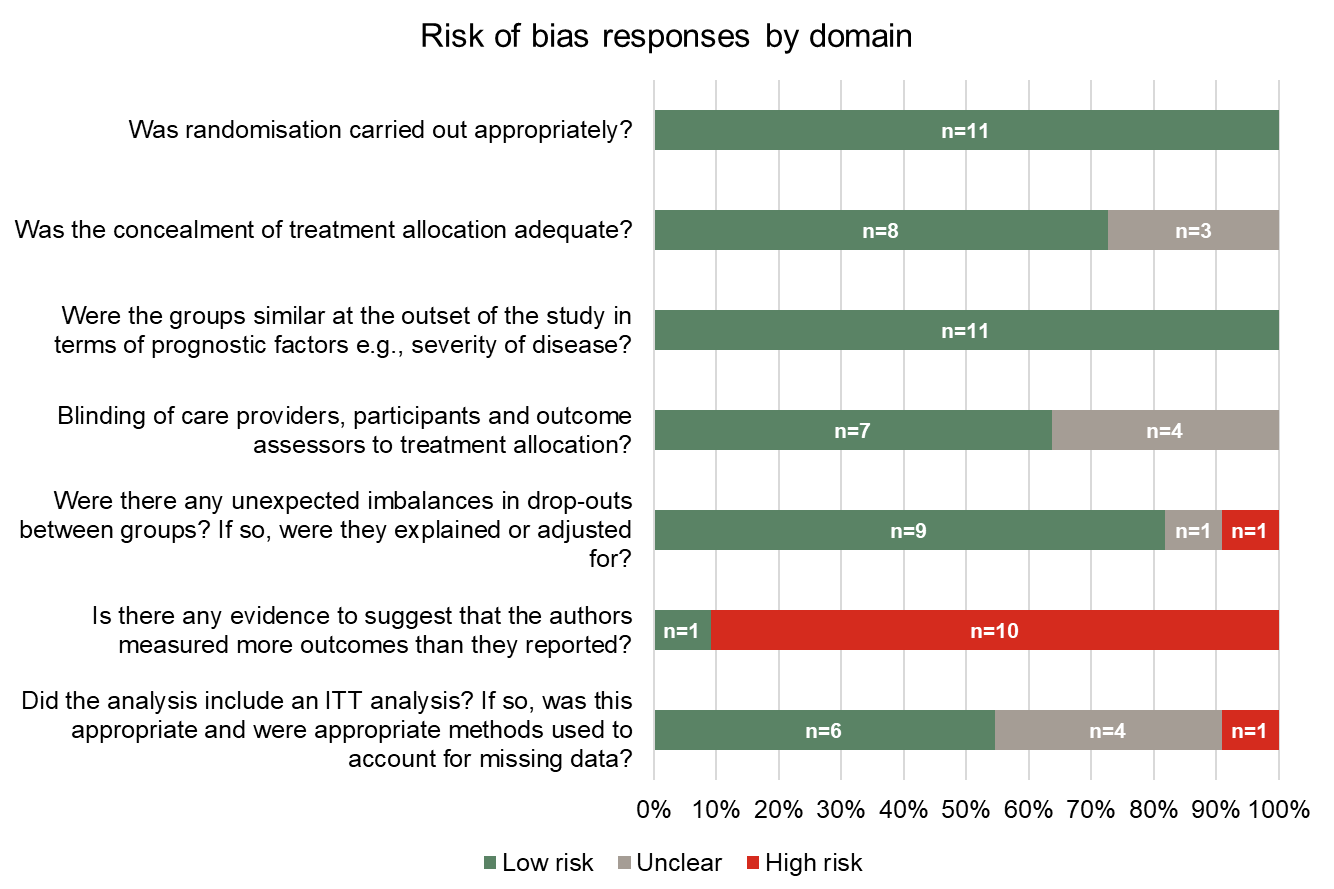


*One study (Study 301 [Lipton et al. 2018]) was available as an abstract only and could not be assessed. An unclear risk of bias was applied when data concerning a particular domain were not provided in the publication. NMA, network meta-analysis; ITT, intention to treat

**Supplementary Figure 2.** Assessment of heterogeneity. Bubble plots showing the treatment effect by placebo response

**(a) Pain freedom at 2 hours**


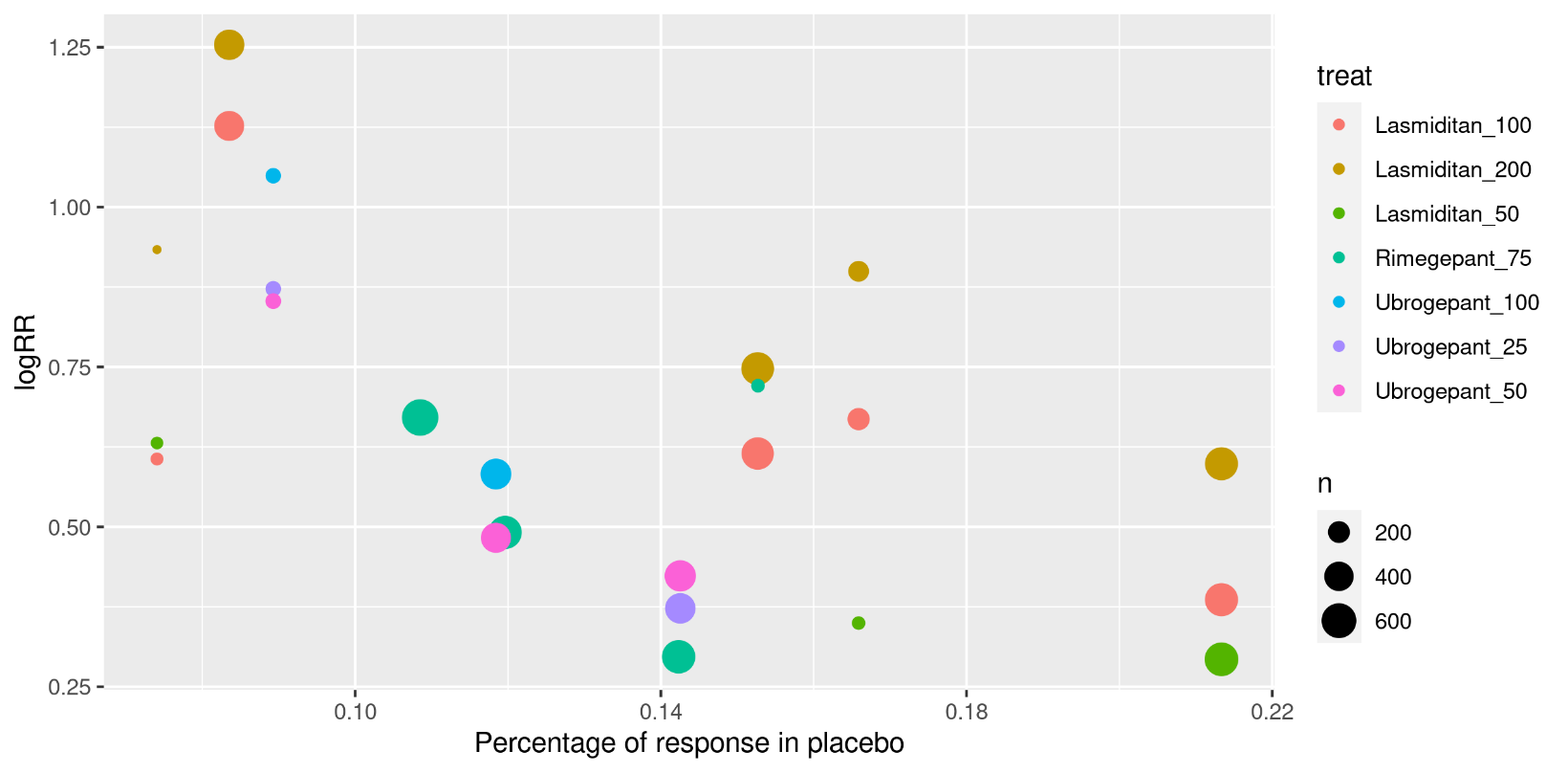


**(b) Pain relief at 2 hours**


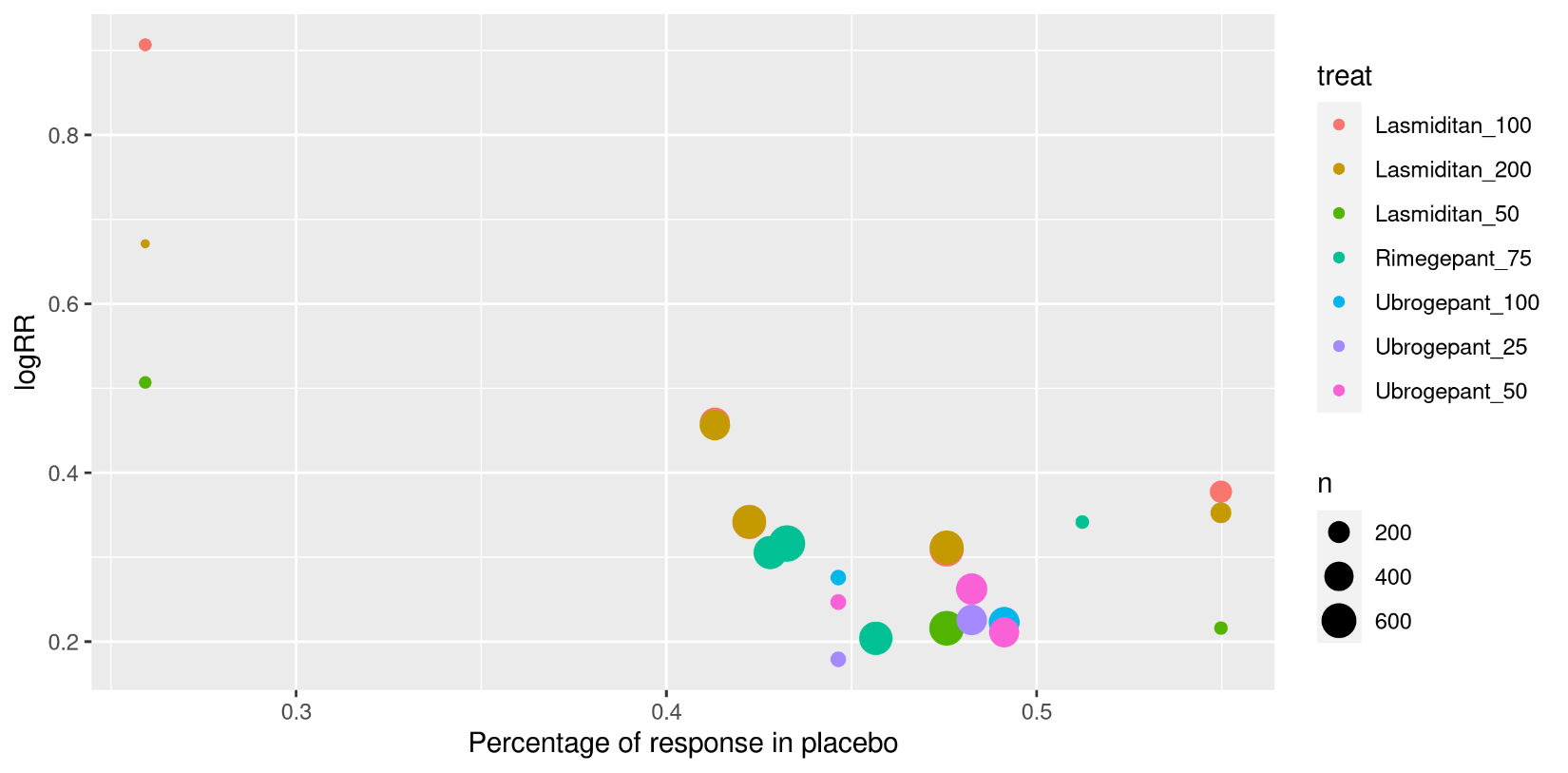


**(c) Pain relief at 1 hour**


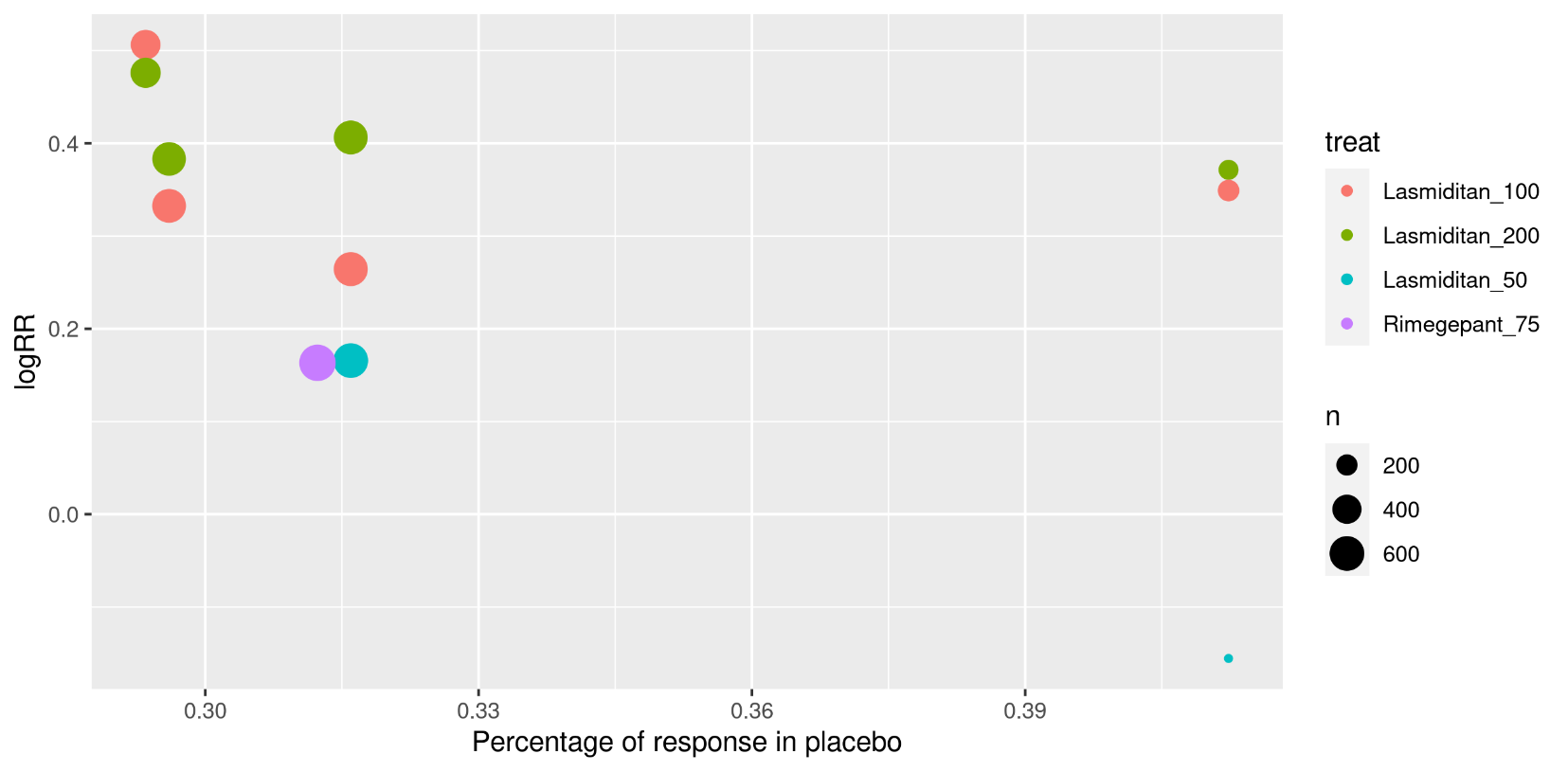


**(d) Sustained pain freedom over 24 hours**


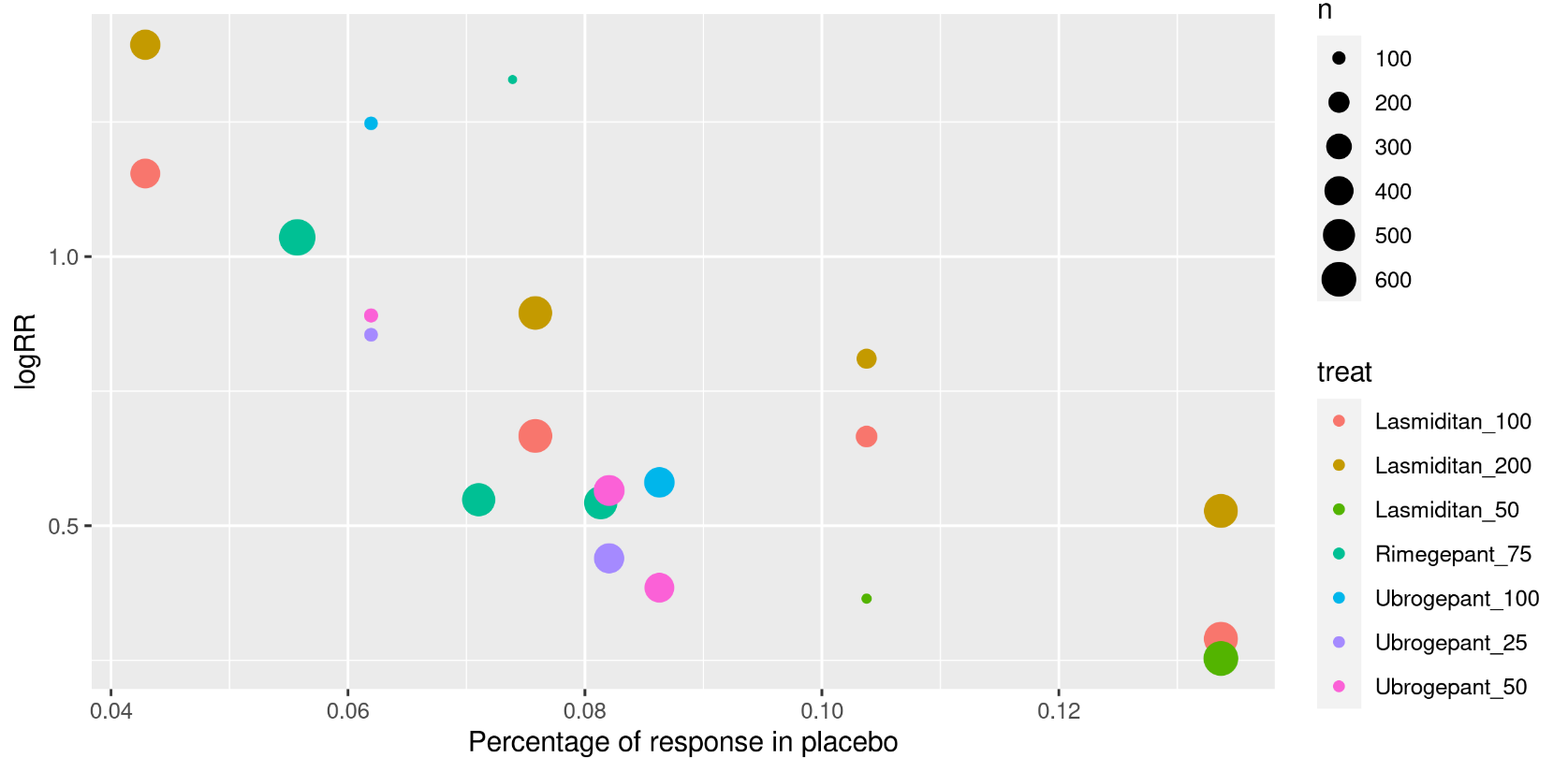


**(e) Most bothersome symptom-free at 2 hours**


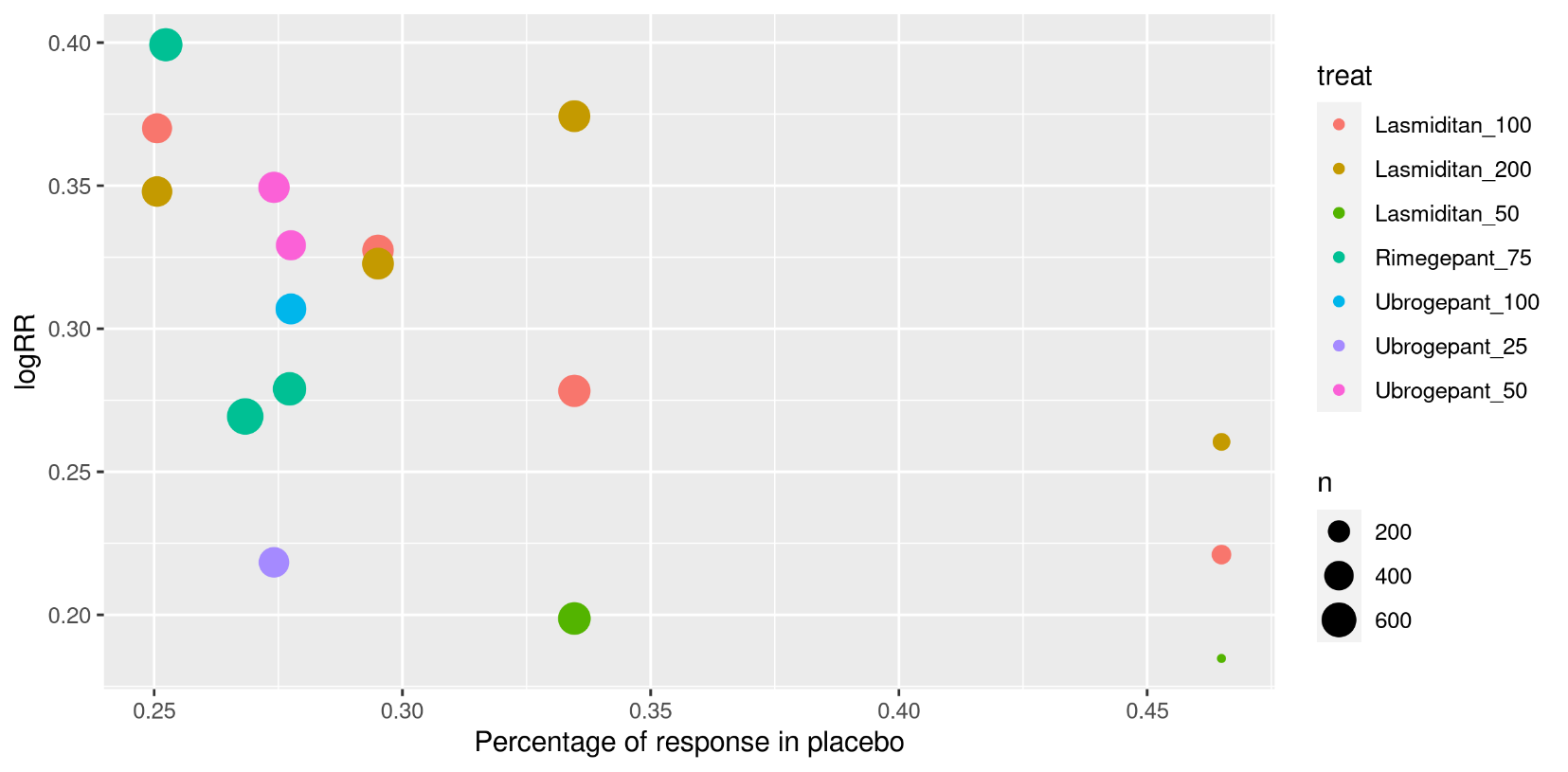


**Supplementary Figure 3.** NMA results for all outcomes – placebo comparisons.


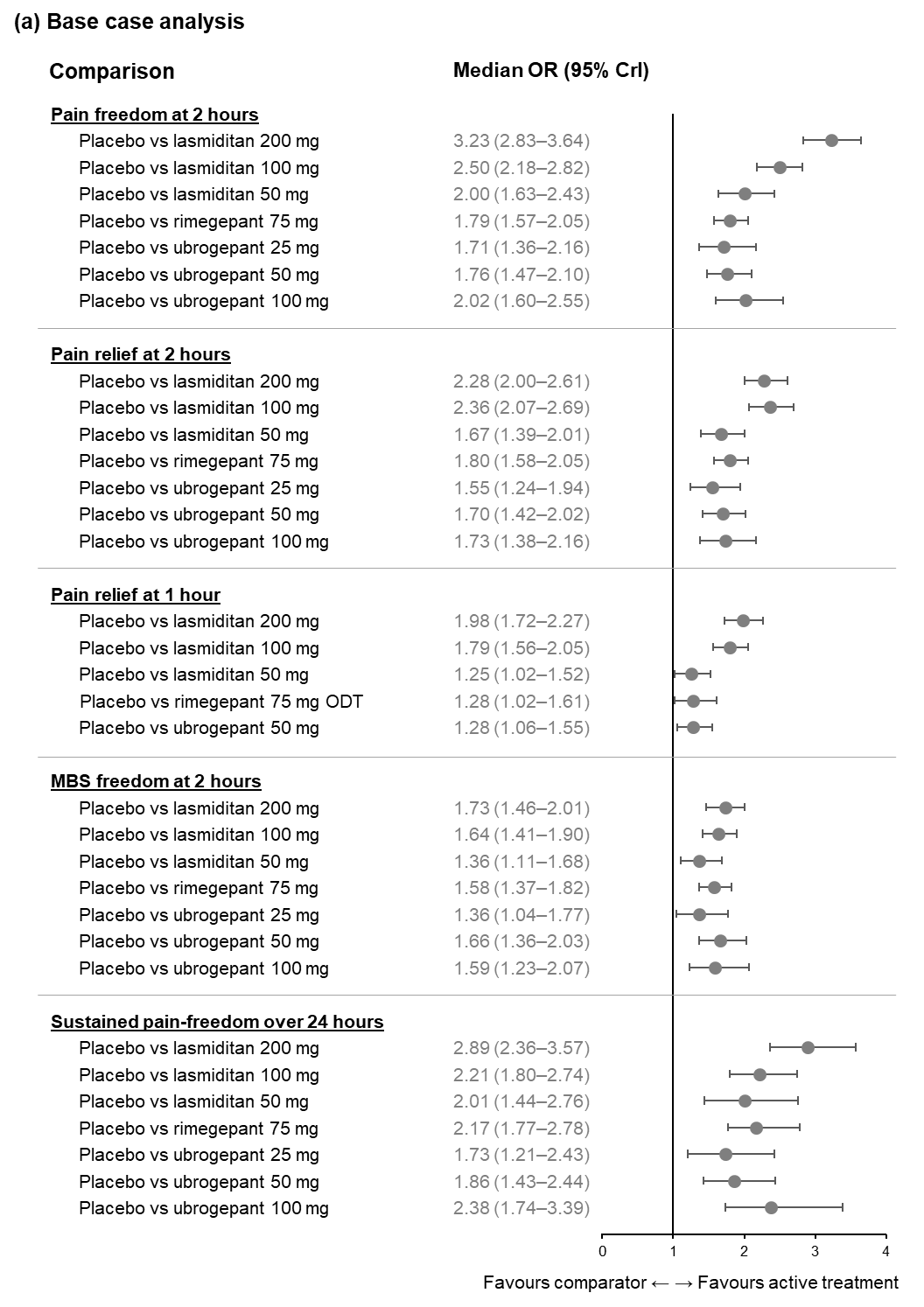


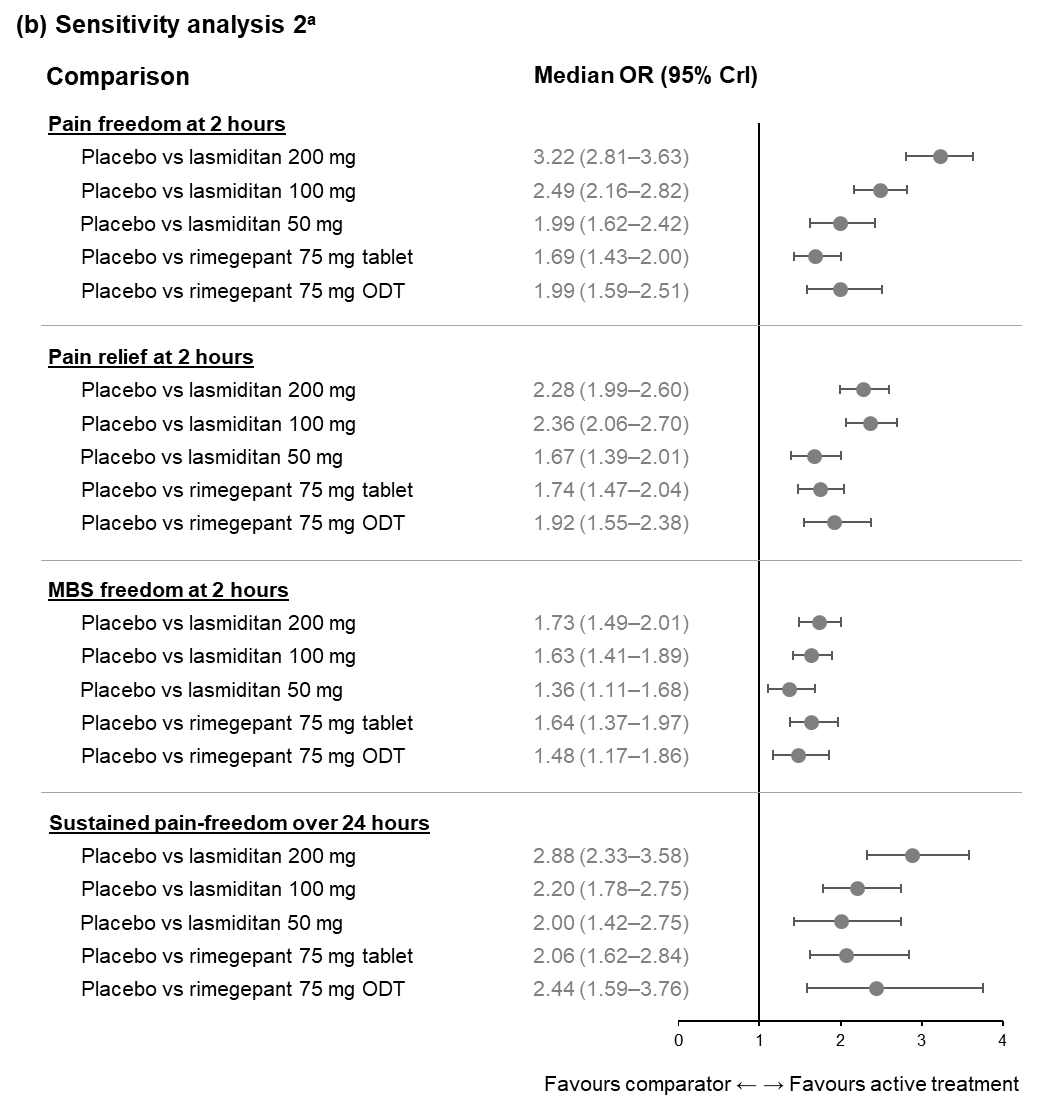


^a^Sensitivity analysis 2 analysed rimegepant according to its mode of administration (tablet or ODT). Pairwise treatment comparisons – results from Bayesian fixed-effects NMA adjusted for baseline risk (**pain freedom at 2 hours:** base case analysis: 36 observations, residual deviance = 36.26 [adjusted baseline risk: mean -0.54 (95% Crl -0.73, -0.28)]; sensitivity analysis 2: 36 observations, residual deviance = 35.74 [adjusted baseline risk: mean -0.52 (95% Crl -0.71, -0.25)]); Bayesian fixed-effects NMA (**pain relief at 2 hours:** base case analysis: 36 observations, residual deviance = 37.31; sensitivity analysis 2: 36 observations, residual deviance = 37.81; **pain relief at 1 hour**: base case analysis: 16 observations; residual deviance = 23.16; **MBS freedom at 2 hours:** base case analysis: 26 observations, residual deviance = 19.07; sensitivity analysis 2: 26 observations, residual deviance = 19.60) or Bayesian random-effects model adjusted for baseline risk NMA (**sustained pain freedom over 24 hours:** base case analysis: 32 observations, residual deviance = 34.00 [adjusted baseline risk: mean -0.72 (95% Crl -1.10, -0.32)]; sensitivity analysis 2: 32 observations, residual deviance = 34.00 [adjusted baseline risk: mean -0.69 (95% Crl -1.10, -0.24)]). Sensitivity analysis 2 could not be performed for pain relief at 1 hour as no suitable data were available for rimegepant tablets. Crl, credible interval; MBS, most bothersome symptom; NMA, network meta-analysis; ODT, oral disintegrating tablet; OR, odds ratio

**Supplementary Figure 4.** Fixed effects analysis with and without baseline risk adjustment for pain freedom at 2 hours.

Pairwise treatment comparisons – results from Bayesian fixed-effects NMA **adjusted for baseline risk** (**base case analysis:** 36 observations, residual deviance = 36.26 [adjusted baseline risk: mean -0.54 (95% Crl -0.73, -0.28)]


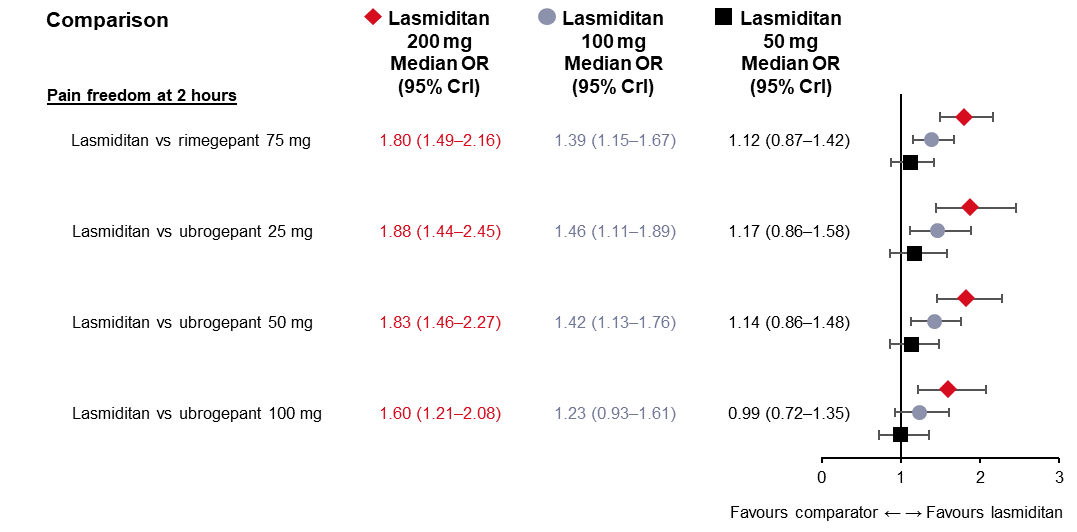


Pairwise treatment comparisons – results from Bayesian fixed-effects NMA **not adjusted for baseline risk** (**base case analysis:** 36 observations, residual deviance = 40.12


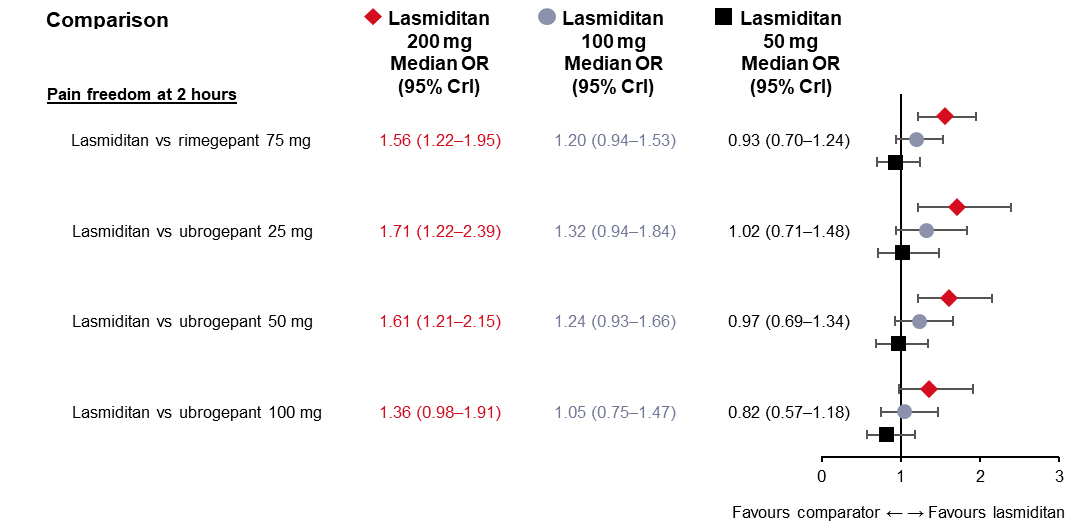


Crl, credible interval; NMA, network meta-analysis; ODT, oral disintegrating tablet; OR, odds ratio

**Supplementary Figure 5.** Sensitivity analysis 1 results for all outcomes.


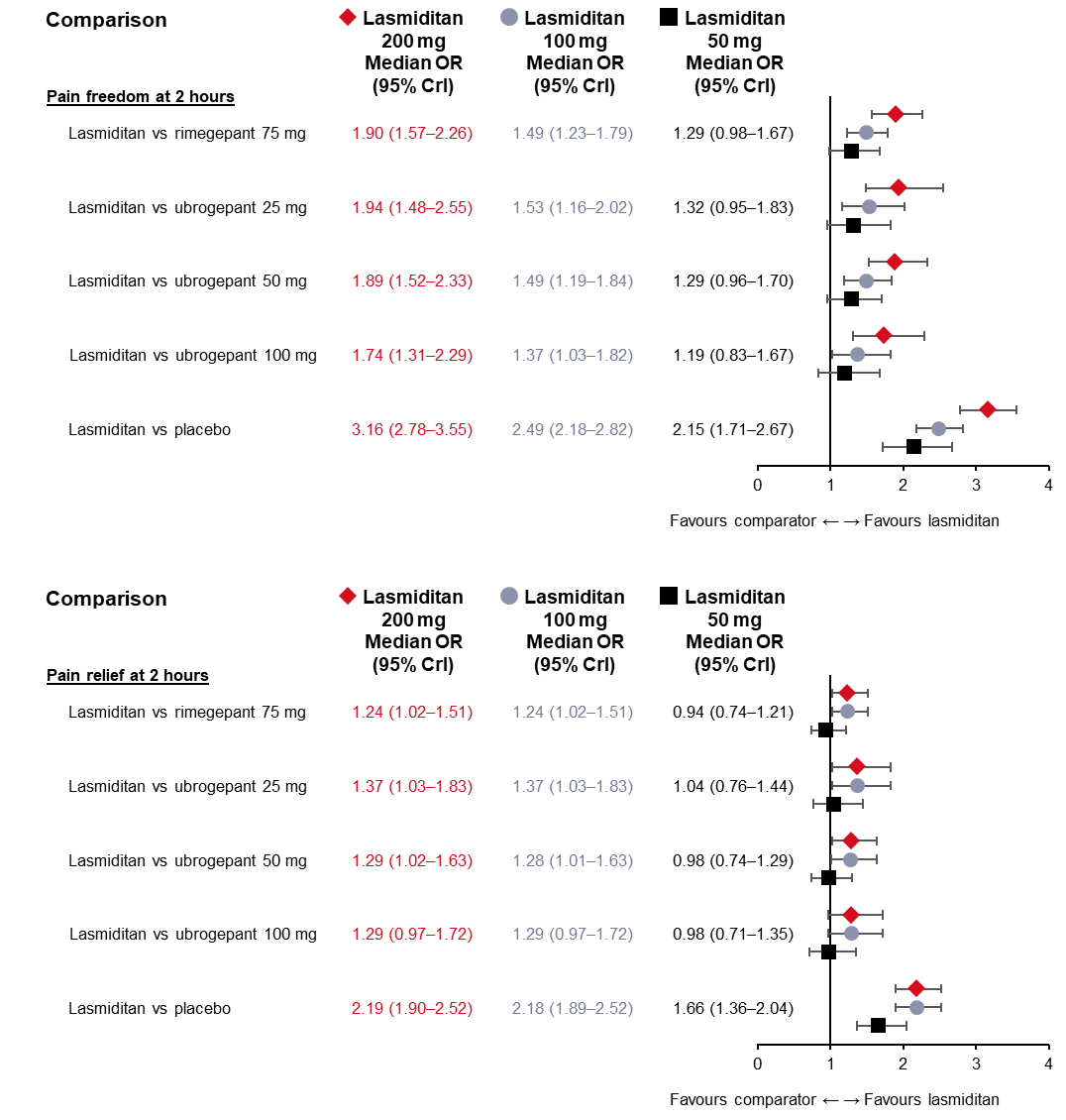


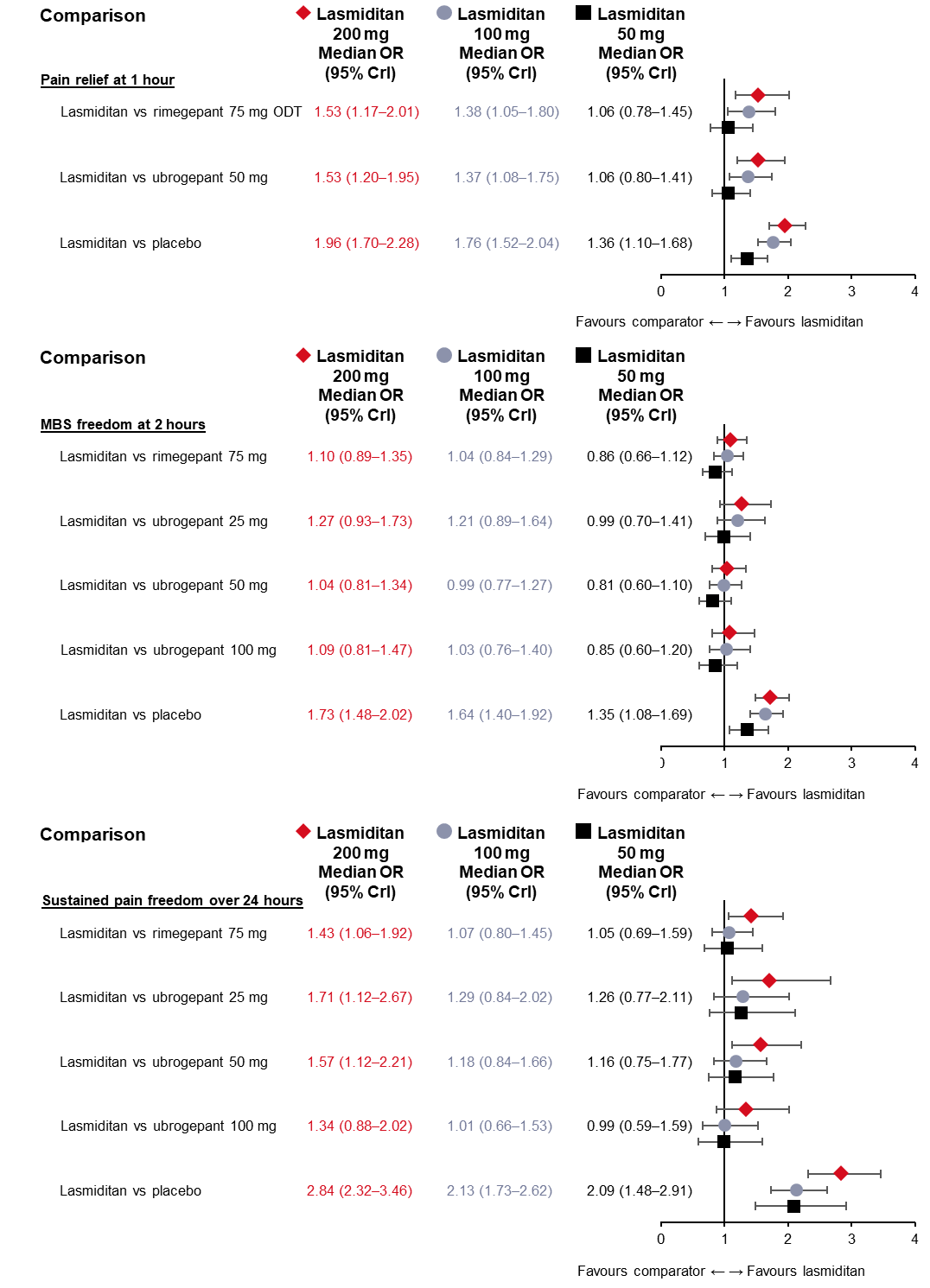


Pairwise treatment comparisons – results from Bayesian fixed-effects model adjusted for baseline risk NMA (**pain freedom at 2 hours:** 22 observations, residual deviance = 20.13 [adjusted baseline risk: mean -0.69 (95% Crl -0.88, -0.44)]); Bayesian fixed-effects NMA (**pain relief at 2 hours:** 22 observations; residual deviance = 21.66; **pain relief at 1 hour:** residual deviance = 15.84; **MBS freedom at 2 hours:** 22 observations; residual deviance = 17.97) or Bayesian random-effects model adjusted for baseline risk NMA (**sustained pain freedom over 24 hours**: 22 observations; residual deviance = 20.5 [adjusted baseline risk: mean -0.77 (95% Crl -1.10, -0.41)]). Crl, credible interval; MBS, most bothersome symptom; NMA, network meta-analysis; ODT, oral disintegrating tablet; OR, odds ratio

**Supplementary Figure 6** NMA results for pain relief at 2 hours.

**
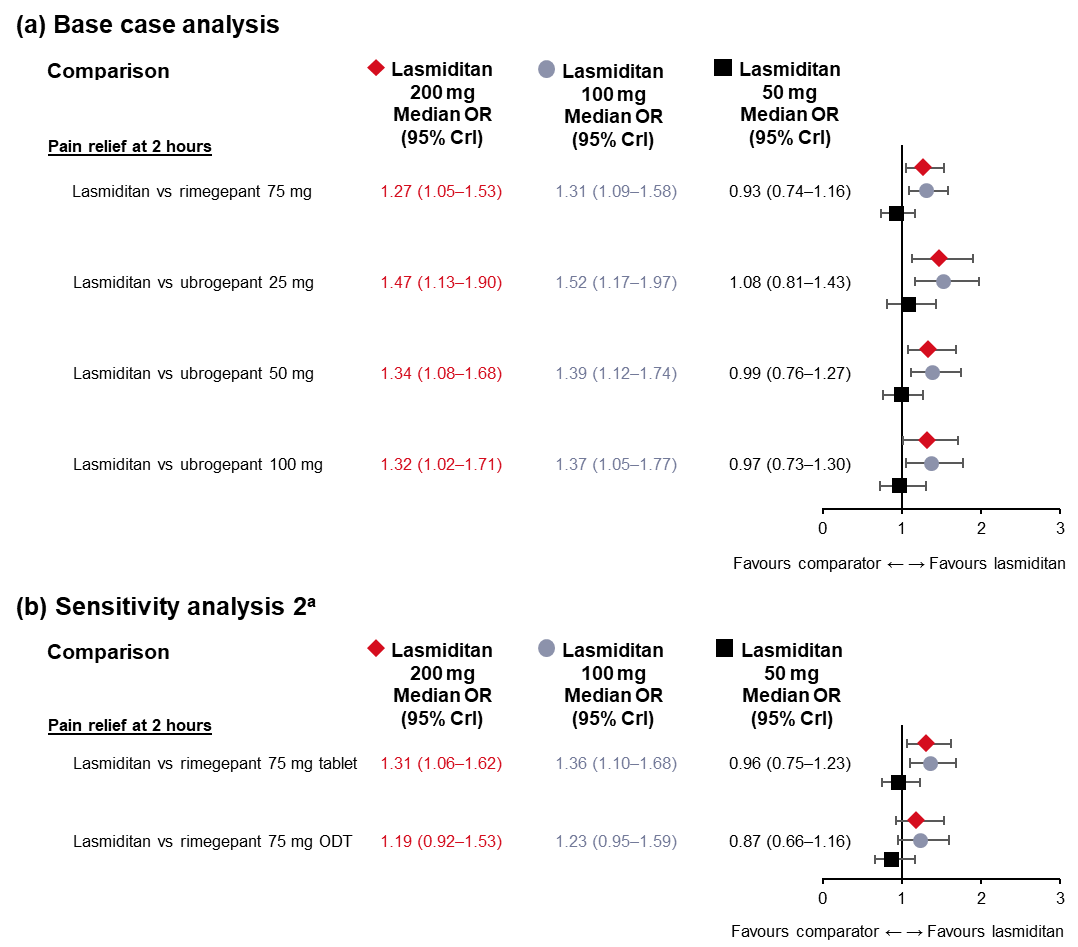
**

^a^Sensitivity analysis 2 analysed rimegepant according to its mode of administration (tablet or ODT). Pairwise treatment comparisons – results from Bayesian fixed-effects NMA (**base case analysis:** 36 observations, residual deviance = 37.31; **sensitivity analysis 2:** 36 observations, residual deviance = 37.81). Crl, credible interval; NMA, network meta-analysis; ODT, oral disintegrating tablet; OR, odds ratio

**Supplementary Figure 7.** NMA results for pain relief at 1 hour – base case analysis.


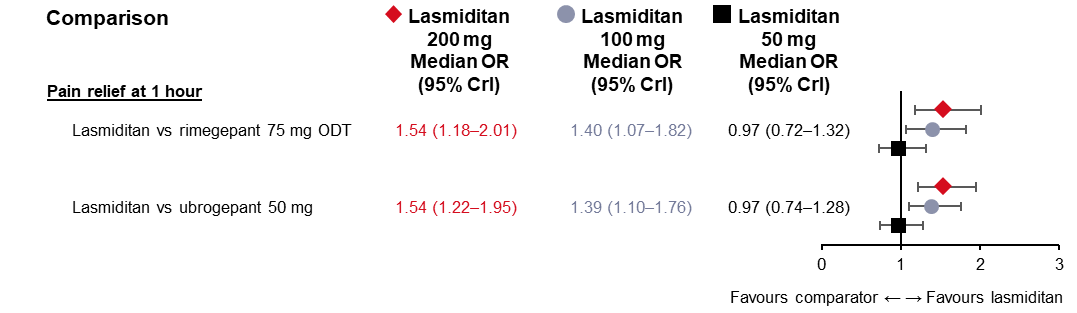


Pairwise treatment comparisons – results from Bayesian fixed-effects NMA (16 observations; residual deviance=23.16). Sensitivity analysis 2 could not be performed for pain relief at 1 hour as no suitable data were available for rimegepant tablets. Crl, credible interval; NMA, network meta-analysis; ODT, oral disintegrating tablet; OR, odds ratio

**Supplementary Figure 8.** NMA results for MBS freedom at 2 hours.


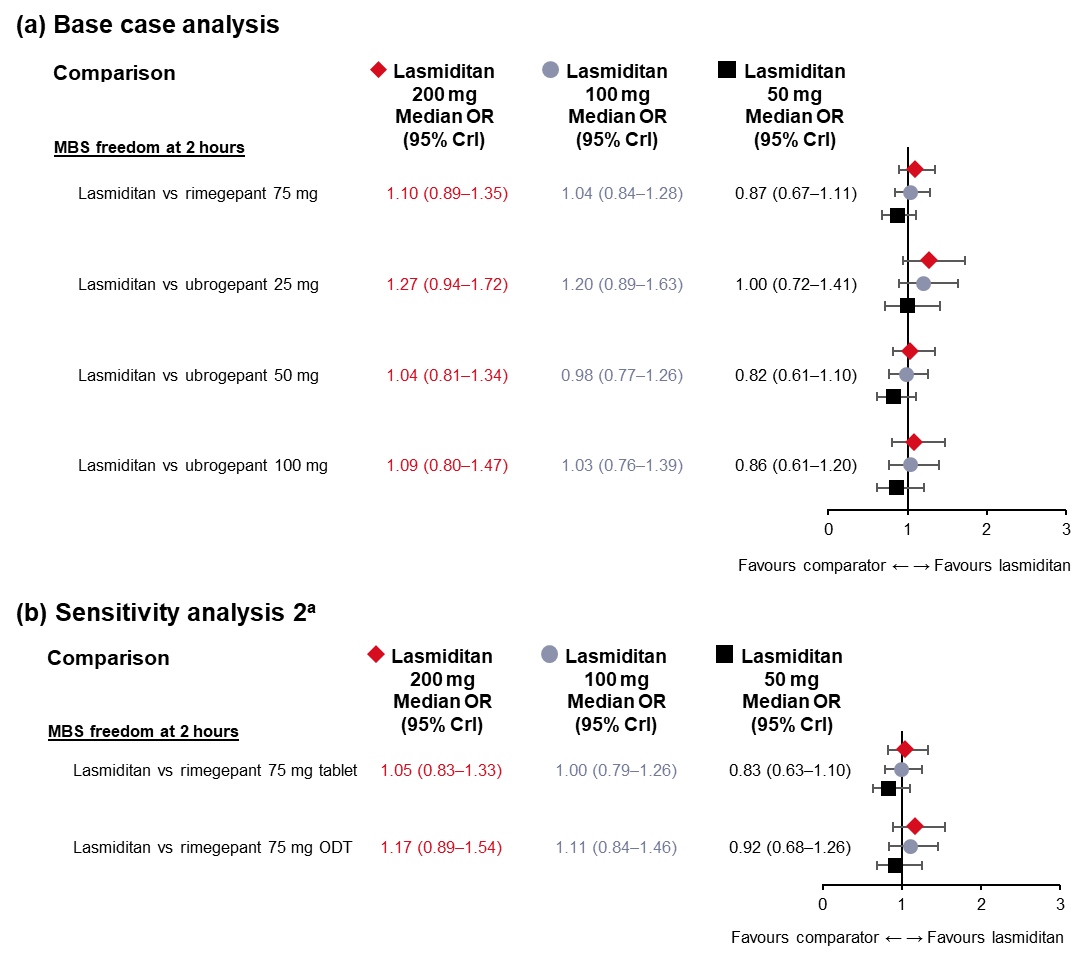


^a^Sensitivity analysis 2 analysed rimegepant according to its mode of administration (tablet or ODT). Pairwise treatment comparisons – results from Bayesian fixed-effects NMA (**base case analysis:** 26 observations, residual deviance = 19.07; **sensitivity analysis 2:** 26 observations, residual deviance = 19.60). Crl, credible interval; MBS, most bothersome symptom; NMA, network meta-analysis; ODT, oral disintegrating tablet; OR, odds ratio

**Supplementary Figure 9.** NMA results for sustained pain freedom over 24 hours.

**
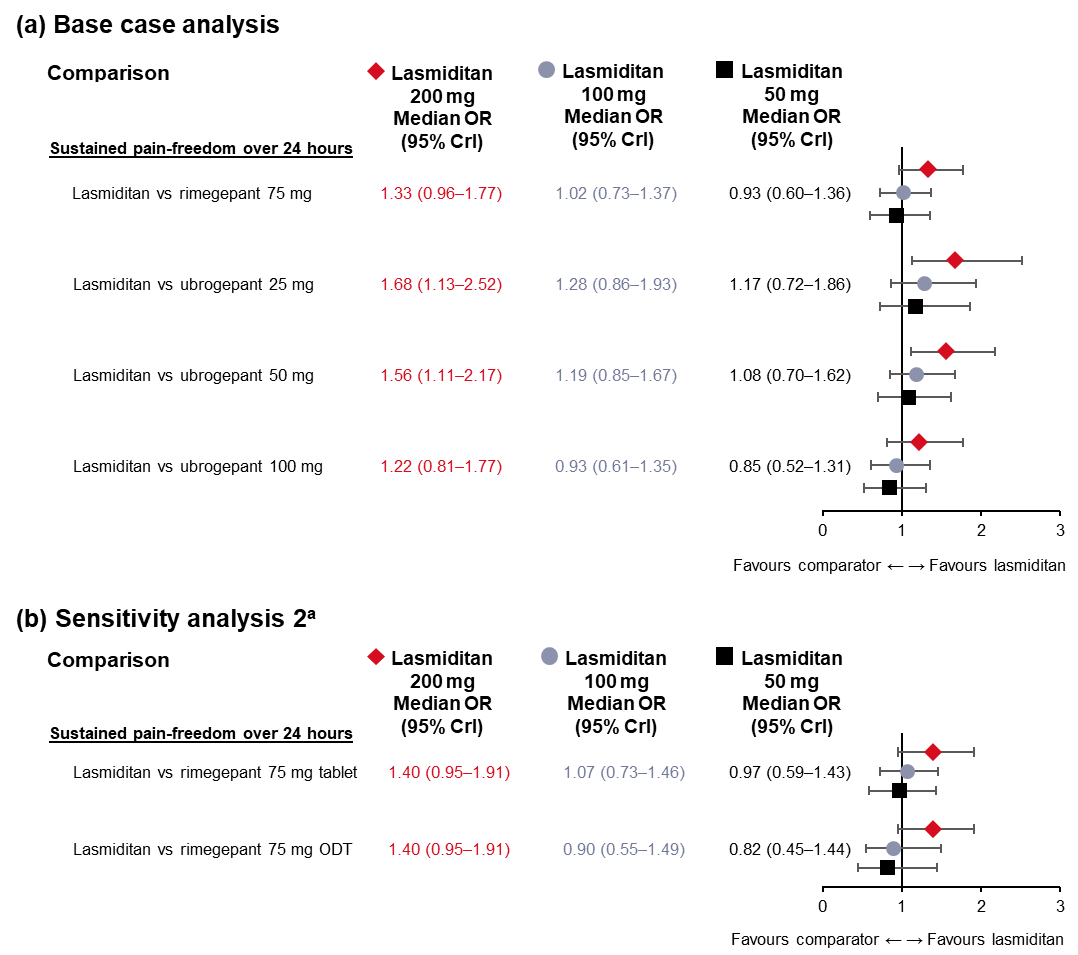
**

^a^Sensitivity analysis 2 analysed rimegepant according to its mode of administration (tablet or ODT). Pairwise treatment comparisons – results from Bayesian random-effects model adjusted for baseline risk (**base case analysis:** 32 observations, residual deviance = 34.00 [adjusted baseline risk: mean -0.72 (95% Crl -1.10, -0.32)]; **sensitivity analysis 2:** 32 observations, residual deviance = 34.00 [adjusted baseline risk: mean -0.69 (95% Crl -1.10, -0.24)]). Crl, credible interval; NMA, network meta-analysis; ODT, oral disintegrating tablet; OR, odds ratio

**Supplementary Figure 10.** Fixed effects analysis with and without baseline risk adjustment for sustained pain freedom at 2–24 hours.

Pairwise treatment comparisons – results from Bayesian random-effects model **adjusted for baseline risk** (**base case analysis:** 32 observations, residual deviance = 34.00 [adjusted baseline risk: mean -0.72 (95% Crl -1.10, -0.32)]

**
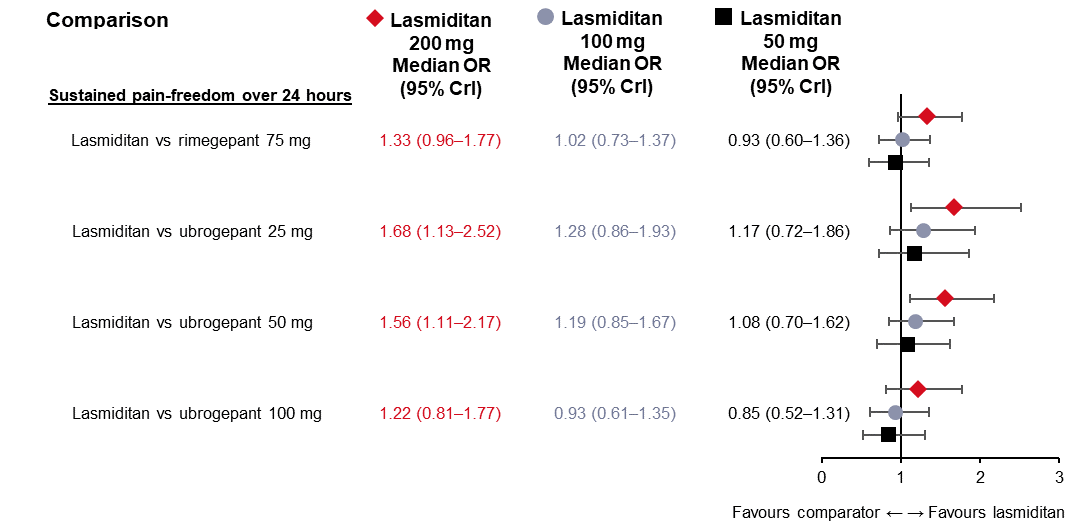
**

Pairwise treatment comparisons – results from Bayesian fixed-effects NMA **not adjusted for baseline risk** (**base case analysis:** 32 observations, residual deviance = 41.41

**
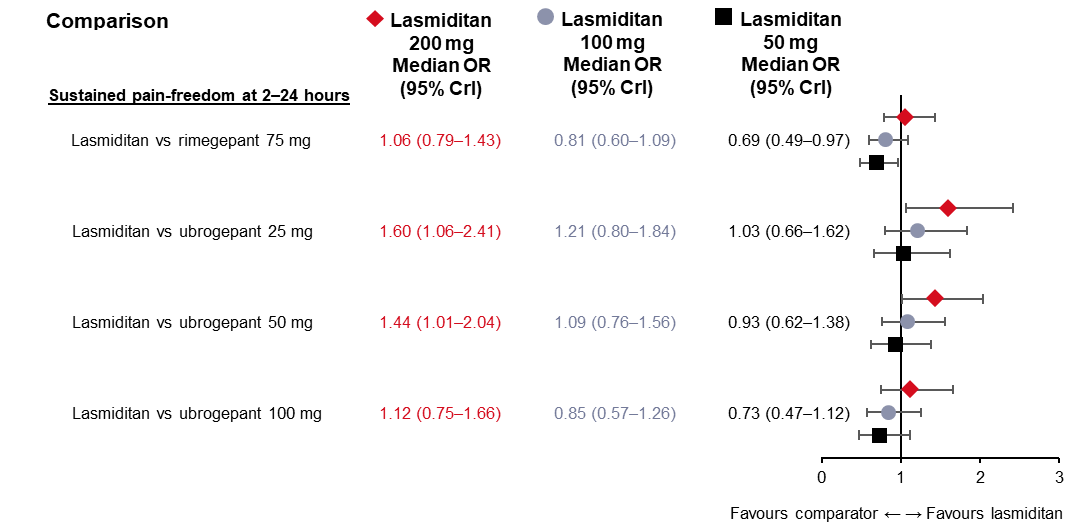
**

Crl, credible interval; NMA, network meta-analysis; ODT, oral disintegrating tablet; OR, odds ratio

**Supplementary Figure 11.** Very early-onset outcomes exploration (sensitivity analysis 3^a^) (base case analysis).


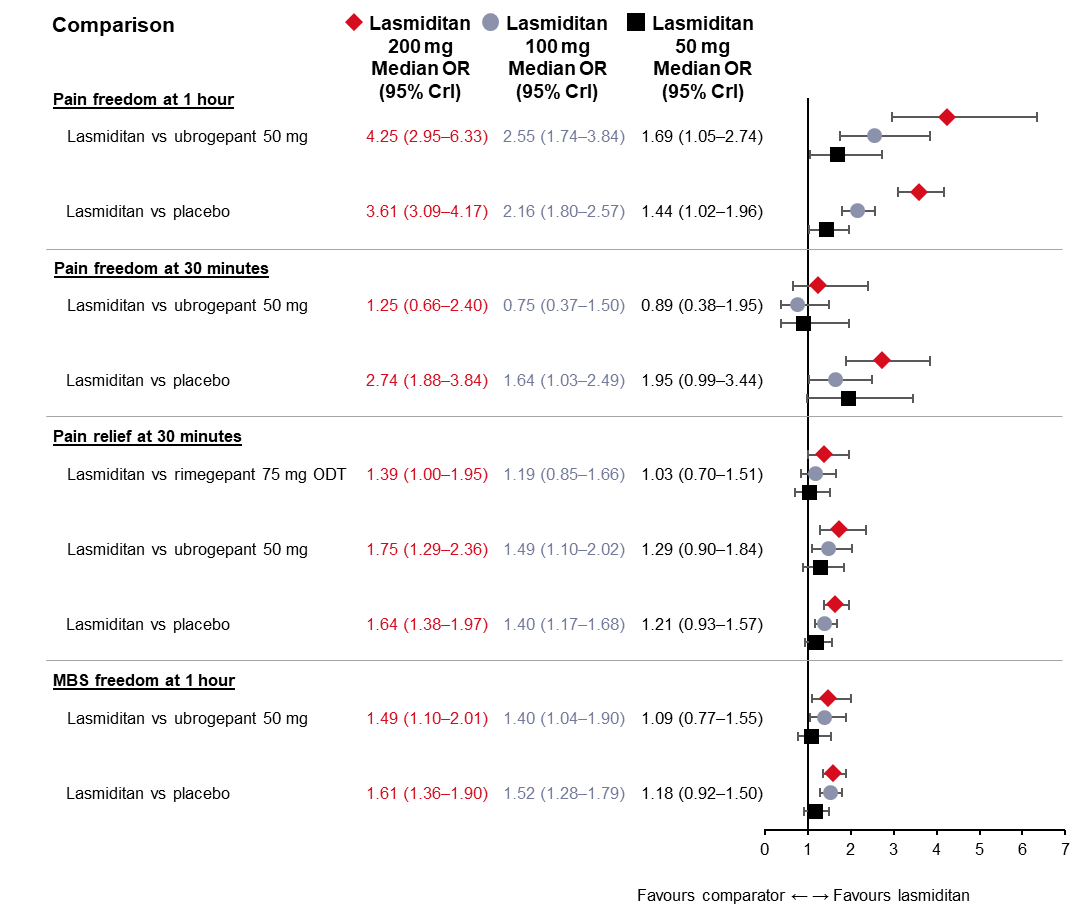


^a^Sensitivity analysis 3 analysed pain freedom at 1 hour and at 30 min, pain relief at 30 minutes and MBS freedom at 1 hour. Assessed using Bayesian fixed-effects model adjusted for baseline risk (pain freedom at 1 hour: 16 observations, residual deviance = 18.45 [adjusted baseline risk: mean -0.79 (95% Crl -0.97, -0.53)]; pain freedom at 30 min: 16 observations, residual deviance = 15.22 [adjusted baseline risk: mean -0.93 (95% Crl -1.45, -0.25)]), Bayesian fixed-effects model (pain relief at 30 min: 18 observations, residual deviance = 14.48; MBS freedom at 1 hour: 16 observations, residual deviance = 17.33). Crl, credible interval; MBS, most bothersome symptom; ODT, oral disintegrating tablet; OR, odds ratio
